# *S100A4* mRNA-protein relationship uncovered by measurement noise reduction

**DOI:** 10.1007/s00109-020-01898-8

**Published:** 2020-04-15

**Authors:** Angelos-Theodoros Athanasiou, Thomas Nussbaumer, Stefan Kummer, Martin Hofer, Iain G. Johnston, Moritz Staltner, Daniela M. Allmer, Milcah C. Scott, Claus Vogl, Joelle M. Fenger, Jaime F. Modiano, Ingrid Walter, Ralf Steinborn

**Affiliations:** 1 Genomics Core Facility, VetCore, University of Veterinary Medicine, Veterinärplatz 1, A-1210 Vienna, Austria; 2 Computational Systems Biology, University of Vienna, Althanstrasse 14, A-1090 Vienna, Austria; 3 VetBioBank, VetCore, University of Veterinary Medicine, Veterinärplatz 1, A-1210 Vienna, Austria; 4 Department of Mathematics, University of Bergen, Bergen, Norway; 5 College of Veterinary Medicine and Masonic Cancer Center, University of Minnesota, 425 East River Road, Minneapolis, MN USA; 6 Institute of Animal Breeding and Genetics, Department for Biomedical Sciences, University of Veterinary Medicine, Veterinärplatz 1, A-1210 Vienna, Austria; 7 Department of Veterinary Clinical Sciences, College of Veterinary Medicine, The Ohio State University, 1900 Coffey Road, Columbus, OH USA; 8 Institute of Pathology, Department of Pathobiology, University of Veterinary Medicine, Veterinärplatz 1, A-1210 Vienna, Austria

**Keywords:** Stably consecutive expressed exons, RT-qPCR data normalization, Quantitative immunohistochemistry, mRNA-protein correlation, Cancer, RNA sequencing

## Abstract

**Abstract:**

Intrinsic biological fluctuation and/or measurement error can obscure the association of gene expression patterns between RNA and protein levels. Appropriate normalization of reverse-transcription quantitative PCR (RT-qPCR) data can reduce technical noise in transcript measurement, thus uncovering such relationships. The accuracy of gene expression measurement is often challenged in the context of cancer due to the genetic instability and “splicing weakness” involved. Here, we sequenced the poly(A) cancer transcriptome of canine osteosarcoma using mRNA-Seq. Expressed sequences were resolved at the level of two consecutive exons to enable the design of exon-border spanning RT-qPCR assays and ranked for stability based on the coefficient of variation (*CV*). Using the same template type for RT-qPCR validation, i.e. poly(A) RNA, avoided skewing of stability assessment by circular RNAs (circRNAs) and/or rRNA deregulation. The strength of the relationship between mRNA expression of the tumour marker S100A4 and its proportion score of quantitative immunohistochemistry (qIHC) was introduced as an experimental readout to fine-tune the normalization choice. Together with the essential logit transformation of qIHC scores, this approach reduced the noise of measurement as demonstrated by uncovering a highly significant, strong association between mRNA and protein expressions of *S100A4* (Spearman’s coefficient *ρ* = 0.72 (*p* = 0.006)).

**Key messages:**

• RNA-seq identifies stable pairs of consecutive exons in a heterogeneous tumour.

• Poly(A) RNA templates for RT-qPCR avoid bias from circRNA and rRNA deregulation.

*• HNRNPL* is stably expressed across various cancer tissues and osteosarcoma.

• Logit transformed qIHC score better associates with mRNA amount.

• Quantification of minor *S100A4* mRNA species requires poly(A) RNA templates and dPCR.

**Electronic supplementary material:**

The online version of this article (10.1007/s00109-020-01898-8) contains supplementary material, which is available to authorized users.

## Introduction

High-quality data quantifying different levels of gene expression are indispensable for the complete understanding of biological processes. Multiple processes beyond transcript concentration contribute to establishing the expression level of a protein. Tumour phenotypes often show comparatively little correlation between mRNA and protein expression levels (Spearman’s correlation coefficient (*ρ*) of ~ 0.50 [1]). In addition to biological variation, various measurement errors may compromise predictability of abundance of a cellular protein from its transcript expression level. For example, a targeted proteomics approach minimized technical error in protein measurement by introducing parallel reaction monitoring of spike-in peptides and a gene-specific RNA-to-protein conversion factor, and hence significantly improved predictability of protein copy numbers from mRNA levels (median Pearson’s *r* of 0.93; [2]).

Here, we aimed at reducing the technical error of transcript expression measurement by RT-qPCR, exemplarily demonstrated for the pathophysiological context of canine osteosarcoma and the metastasis-promoting S100A4 protein*.* Osteogenic sarcoma, or osteosarcoma, represents a cancerous bone tumour that is rare, spontaneous, aggressive and malignant, produces an osteoid matrix and appears in six subtypes or their combinations [3]. A comparative approach to studying osteosarcoma has highlighted many clinical and biological aspects of the disease that are similar between dogs and humans. On the other hand, there are important species-specific differences that are becoming increasingly recognized [4]. The comparative nature of the orthologous diseases of man and dog is supported by shared findings such as the higher odds of metastasis determined for trunk osteosarcomas at diagnosis compared to those with lower limb osteosarcomas [5], broad genomic similarity with recurrent copy number aberrations in oncogenes and tumour suppressor genes such as *MYC*, *CDKN2A/B*, *RB1* and *PTEN* [6], low point mutation burden, high structural complexity, frequent mutations in *TP53*, *PI3K* and *MAPK* pathways and low expression of immune-associated genes [7], common starting genes of chimeric transcripts [8] as well as some overlapping transcriptional programs [9].

Our molecular target, S100A4, is a member of the S100 protein family that is expressed in a variety of cells, such as stem cells, fibroblasts, neutrophils, monocytes, lymphocytes and malignant cells. Like many other members of the S100 family, it shows intracellular and extracellular localization [10]. Under non-pathological conditions the protein is mostly localized inside the cell [11]. Noncancerous inflammatory indications as well as metastatic progression of cancer are accompanied with increased translocation of the protein into the extracellular space [10].

Here, technical error in RT-qPCR measurement, which is challenged by the extensive genetic and expression heterogeneity of the tumour condition, was reduced by the following critical parameters. The first was the generation of mRNA-Seq data for the study condition and selection of stably expressed sequences at exon-level resolution. The second was the consistent use of poly(A) RNA templates across both steps of the analysis pipeline, i.e. selection and verification of exon sequences by mRNA-Seq profiling and RT-qPCR. In addition, a nonlinear transformation commonly used to transform proportion data in other fields of science and economy was extended to qIHC analysis. Logit transformation of qIHC proportion data approximated normality with little variation in variances, such that powerful linear models could be applied. Together, the improvements revealed the association between the number of mRNA copies of the *S100A4* gene and the abundance of the translated protein expressed as qIHC score.

## Materials and methods

### Biological material

Normal canine osteoblasts (Cell Applications Inc., San Diego, CA, USA) were cultured in a canine osteoblast medium (Cell Applications Inc.).

Samples of spontaneously developed osteosarcoma were collected from large- and medium-sized dog breeds during routine medical treatment at the College of Veterinary Medicine of the University of Minnesota or at the Animal Hospital of the University of Veterinary Medicine of Vienna, following the rules of the local ethical committees. For transcript profiling by mRNA-Seq or RT-qPCR, experimental specimens were grouped into two cohorts, termed set 1 and set 2, respectively.

Set 1 included ten osteosarcoma tissues (#0320, #0460, #1033, #1091, DOS-8, DOS-71, DOS-73, DOS-119, DOS-126 and DOS-127) and five primary cell lines (OSCA-8, OSCA-30, OSCA-32, OSCA-40 and OSCA-78) derived from newly diagnosed patients prior to treatment with cytotoxic chemotherapy drugs (Table S1).

Set 2 comprised exclusively tumour tissue (*n* = 13, Table S2). Clinical and pathological data of the sample cohort are listed in Tables S1 and S2, respectively. Aliquots of osteosarcoma tissue were snap-frozen at − 170 °C or preserved in an RNA-stabilizing buffer (RNAlater; Qiagen, Hilden, Germany) and stored in the gas phase over liquid nitrogen.

### Short tandem repeat DNA profile analysis for cell line authentication

To exclude cross-contamination and other causes of misidentification, the five osteosarcoma cell lines used in this study were genotyped at 15 short tandem repeat (microsatellite) loci. Profiling of microsatellites was performed by IDEXX BioResearch (www.idexxbioresearch.com). For authenticating genetic relatedness between donor (tumour tissue or original cell line) and the cell line at the respective passage used for this study, we adopted a threshold of at least 80% matching alleles [12]. This percentage match criterion was based on the idea that the microsatellite profile from a malignant tissue can vary with loss of heterozygosity and an elevated incidence of microsatellite instability and complexity, and allowed for some genetic drift with increasing passage number of a cell line. The match value was obtained by dividing the number of shared alleles by the total number of alleles in the questioned profile.

Our authenticating genotype data included one to two deviating microsatellite alleles, mostly loss of heterozygosity rather than a change in the allele length (Data S1).

### Extraction of total RNA

RNA of normal osteoblasts and a part of the tissues of sample set 1 was isolated using the TRIzol™ Reagent, a monophasic solution of phenol and guanidine isothiocyanate (Thermo Fisher Scientific, Waltham, MA, USA). RNA of tissues of set 2 and cultured osteosarcoma cells of set 1 was extracted with a silica-based membrane combined with micro-spin technology.

The concentration of RNA subjected to mRNA-Seq analysis was measured using the fluorescent dye-based Quant-iT™ RiboGreen® RNA Assay Kit (Thermo Fisher Scientific). Its integrity was assessed by capillary electrophoresis on the 2100 Bioanalyzer instrument (Agilent Technologies, Santa Clara, CA, USA). Next-generation sequencing libraries were prepared only from samples that exhibited an RNA Integrity Number (RIN) value of at least 8 and a quantity higher than 1 μg.

Concentration and purity of RNAs subjected to profiling by RT-qPCR were determined on the NanoDrop™ 2000c spectrophotometer (Thermo Fisher Scientific). Minimum RNA intactness assessed at the 4200 TapeStation System (Agilent Technologies) was set at a value of ≥ 6.4 RNA integrity number equivalent (RIN^e^).

### Transcriptome profiling by mRNA-Seq

Sequencing libraries focused on poly(A) RNA were generated from 1 μg total RNA using the TruSeq RNA library preparation kit v2 (Illumina, San Diego, CA, USA) and sequenced with sequencing-by-synthesis technology on the HiSeq™ 2000 Sequencing System (Illumina).

Primary analysis and demultiplexing of mRNA-Seq data was performed using the CASAVA software version 1.8.2 (Illumina). For hierarchical clustering of expression patterns, sequence reads were aligned to the reference genome of the dog (*Canis lupus familiaris*) or the dingo (*Canis lupus dingo*) using the genome assemblies CanFam3.1 or ASM325472v1, respectively, downloaded from the Ensembl genome browser and the Spliced Transcripts Alignment to a Reference (STAR) software version 2.7.3a ([13]; https://github.com/alexdobin/STAR/releases).

For selection of stable exons, we used TopHat (https://ccb.jhu.edu/software/tophat/index.shtml), another popular, but less recent splice-aware aligner.

### Expression stability ranking of single and neighbour exons based on *CV*

The stability of exonic sequences in the mRNA-Seq expression data was ranked in ascending order based on the *CV,* defined as the ratio of the standard deviation to the mean. Individual *CV* values were calculated for single exons and three exon-neighbour combinations, namely, a pair of direct neighbours (*i* and *i* + 1), a trio of direct neighbours (*i*, *i* + 1 and *i* + 2) and an exon paired with its neighbour after next (*i* and *i* + 2). Calculation was performed using R package version 3.5.5 (www.R-project.org/).

### Enrichment analysis for gene ontology (GO) terms

The most stable genes were annotated by one or more GO terms using the Blast2Go tool (www.blast2go.com). Briefly, Standard Protein BLAST (https://blast.ncbi.nlm.nih.gov/Blast.cgi?PAGE=Proteins) was performed against the non-redundant database of NCBI for all mammals (taxon 40674). Only the first 20 alignments passing the *E* value cut-off of 1.0E-3 with a coverage of at least 90% against the subject sequences were considered for annotation. The GO terms retrieved by InterPro scanning at the web server of the European Bioinformatics Institute (www.ebi.ac.uk/interpro/) were converted and merged with the annotation. The annex function was employed to assign the GO terms obtained by GO mapping to the query sequences. Finally, “slimming” was carried out to identify the most representative biological processes that were subsequently subjected to enrichment analysis. The annotations of all known canine proteins were used as a background list to create a 2 × 2 contingency table for calculating the Fisher’s exact test in Microsoft Excel (https://udel.edu/~mcdonald/statfishers.xls). Enriched GO terms with a false discovery rate of less than 0.01 were reported. Concordance of the enrichment results was evaluated at the GeneMANIA database (http://genemania.org) using human genomics and proteomics data as orthologous substitute for the dog and the “GO biological process” as the weighting method.

### RT-qPCR

Total cellular RNA or its poly(A)-RNA fraction extracted with paramagnetic oligo (dT)_25_ beads of the NEBNext® Poly(A) mRNA Magnetic Isolation Module (New England Biolabs, Ipswich, MA, USA) was used as the template for cDNA synthesis.

First-strand cDNA was synthesized in duplicate using a reaction volume of 20 μl and the Transcriptor High Fidelity cDNA Synthesis Kit (Roche Life Science, Vienna, Austria). The reaction contained 1 × Transcriptor RT reaction buffer, 20 U Protector RNase inhibitor, 1 mM dNTP mix, 60 μM random hexamers or 2.5 μΜ oligo(dT)_18_ primer in case of poly(A)-RNA templates, 10 U Transcriptor Reverse Transcriptase and 500 ng total RNA or 500 pg poly(A) RNA.

Putative contamination with genomic DNA was monitored by a reaction lacking reverse transcriptase. RT was performed at 55 °C for 60 min and terminated at 85 °C for 5 min using the MJ Research PTC-200 Thermal Cycler (Bio-Rad, Hercules, CA, USA). The undiluted cDNAs were stored at − 20 °C until further analysis.

Oligonucleotide sequences of the qPCR assays (Table S3) were designed using the program Primer Express 2.0 (Applied Biosystems, Foster City, CA, USA). The chance of amplifying co-isolated genomic DNA was reduced by designing a PCR product that spanned an exon-intron boundary or flanked an intron of at least 750 bp. Putative primer dimerization was evaluated with NetPrimer (Premier Biosoft International, Palo Alto, CA, USA; www.premierbiosoft.com/netprimer/). The secondary structure of the PCR amplicon was predicted on the Mfold Web Server (http://unafold.rna.albany.edu/?q=mfold/DNA-Folding-Form). Amplicon specificity was evaluated by NCBI's Primer-Blast (www.ncbi.nlm.nih.gov/tools/primer-blast/) using the “non-redundant” database of the dog (taxid number 9615).

The qPCR performed in a volume of 15 μl was composed of 1 × PCR buffer B2 (Tris-HCl, (NH_4_)_2_SO_4_ and Tween-20; Solis Biodyne, Tartu, Estonia), 1 × dNTP mix that partially replaced dTTP with dUTP for putative removal of carry-over contamination by uracil DNA glycosylase (0.2 mM of each dATP, dCTP and dGTP, 0.08 mM of dUTP and 0.12 mM of dTTP; Solis Biodyne), 0.4 × EvaGreen I dye (Biotium, Fremont, CA, USA) or 200 nM hydrolysis probe (Integrated DNA Technologies, Leuven, Belgium) depending on the assay, 3.5 mM MgCl_2_, 200 nM of each primer, 1 U HOT FIREPol® DNA polymerase (Solis Biodyne) and 1.5 μl or 6 μl of diluted cDNA. For target quantification in total cellular RNA or poly(A)-RNA templates, cDNAs were ten- or sixfold diluted, respectively. All qPCR assays that were run in duplicates (or triplicates in case of the *S100A4* transcript variants) included a minus-RT control and a no-template control to rule out cross-contamination of reagents and surfaces. Amplification and monitoring of fluorescence were performed on the Corbett Rotor-Gene 6000 Real Time PCR System (Qiagen) operated by the software version Rotor-Gene Q 2.1.0.9. Cycling conditions consisted of an initial 15-min incubation step at 95 °C for polymerase activation and template denaturation, followed by 50 cycles of 95 °C denaturation for 15 s, 60 °C annealing for 20 s and 72 °C elongation for 20 s. Finally, a dissociation curve was recorded over the range of 60 to 95 °C at increments of 1 °C every 5 s. In case of the probe-based qPCR format, amplification was achieved over 50 cycles consisting of a 15-s denaturation step at 95 °C and combined annealing and elongation for 60 s at 60 °C. A qPCR assay was considered unaffected by co-amplification of genomic DNA if the minus-RT control produced an efficiency-adjusted Δ*Cq* value of at least 5.

In addition to the amplicon melting profile, specificity of amplification primers was validated by electrophoresis on a 1% agarose gel (Fig. S1) using 1 × sodium borate buffer (10 mM NaOH, pH adjusted to 8.5 with boric acid), 8.75 μl GelGreen™ Nucleic Acid Stain (Biotium) per 100 ml gel and the “100-bp DNA Ladder” (Solis Biodyne) for determination of DNA fragment size.

### Determination of qPCR amplification efficiency and outlier treatment

For target sequences of at least moderate abundance, efficiency of qPCR amplification (*E*) can be determined from raw fluorescence data without the need of a standard curve. For each well, an individual efficiency was calculated from the exponential phase of the raw (i.e. not baseline-corrected) amplification curve using the Real-time PCR Miner (http://ewindup.info/miner/). The mean of individual efficiencies, *E*_*fi*_, served to adjust *Cq* values measured at an amplification efficiency of less than 100% according to the term *Cq* × log_10_ (*E*_*fi*_ + 1)/log_10_(2). Following efficiency correction of *Cq* values, outliers from quadruplicate RT-qPCR measurements, i.e. qPCR replicates for both cDNA duplicates, were identified and handled as follows. In case of quadruplicate *Cq* values, we removed the technical replicate that caused a standard deviation of more than 0.5 cycles. If the means of the qPCR duplicates run for each of the two cDNA replicates differed by more than one cycle, the cDNA replicate that exhibited the highest deviation from the global average of the sample cohort was removed from analysis (*n* = 7, validation of reference exons by RT-qPCR).

### RT-qPCR measurement of exon’s expression stability

The two steps of an RT-qPCR assay were run in duplicates starting with the RT reaction. The average of the duplicate’s raw *Cq* values was efficiency-adjusted and assessed for expression stability using four common statistical algorithms, geNorm implemented in the qbase+ software 3.1 (www.qbaseplus.com), the Microsoft Excel-based programs NormFinder (https://moma.dk/normfinder-software) and BestKeeper (www.gene-quantification.de/bestkeeper.html) as well as the Comparative Δ*Cq* method accessed via the web-based tool RefFinder (www.heartcure.com.au/reffinder/). The ranks of the individual algorithms were aggregated into a final weighted rank using the R package RankAggreg version 3.4.4 for Windows (https://CRAN.R-project.org/package=RankAggreg), run with the implemented cross-entropy Monte Carlo algorithm with Spearman’s footrule distance. The R code for rank aggregation analysis is provided as File S1.

The minimum gene set for RT-qPCR normalization was determined by the geNorm algorithm. The software sorts genes in ascending order according to their expression stability measure (*M*), computes a *NF* for each gene set using the geometric mean of their expression values and determines the optimal number of normalizers based on pairwise variation (*V*_*n*_*/V*_*n + 1*_) between two sequential factors, *NF*_*n*_ and *NF*_*n* + *1*_. The common cut-off value of *V*_*n*_*/V*_*n + 1*_ < 0.15 determined the lowest number of genes for accurate normalization. This minimum gene number was adopted to compose the *NFs* for the other stability algorithms. In another round of stability assessment with the RankAggreg package, we computed consensus ranks for the single genes and the added four *NF*s.

Co-regulated gene relationship of the gene pair composing the best *NF* was evaluated based on information for the dog contained in version 7.3 of the gene co-expression database COXPRESdb (http://coxpresdb.jp).

### In silico analysis of alternative polyadenylation in the 3’ untranslated region (UTR) of canine *S100A4* mRNA

Hexanucleotide motifs signalling canonical (AAUAAA) as well as non-canonical (AUUAAA, AGUAAA or UAUAAA) polyadenylation were used as query sequences for in silico analysis by “Poly(A) Signal Miner” integrated in DNA functional site miner (http://dnafsminer.bic.nus.edu.sg).

### Counting *S100A4* mRNA copies by digital PCR (dPCR)

The copy number concentration of a cDNA target sequence was determined by dPCR on the Applied Biosystems QuantStudio™ 3D Digital PCR System (Thermo Fisher Scientific) using the QuantStudio™ 3D Digital PCR MasterMix and oligonucleotide sequences developed in this work (Table S3). The highest expressor of *S100A4* mRNA (sample #2097) was used for “translating” the sample’s *Cq* values measured by the various *S100A4* RT-qPCR assays into numbers of transcript copies.

### Assignation of biochemical processes using gene ontology classification

Molecular functions of the gene pair regarded as the most appropriate *NF* were derived from the Gene Ontology Database (release: 1st of January 2019; http://amigo.geneontology.org/amigo).

### Quantitative immunohistochemistry (qIHC) for S100A4

The area fraction of (brown) colour pixels resulting from immunohistochemical staining against S100A4 was automatically scored by quantitative image analysis.

Paraffin sections were rehydrated and blocked by 1.5% normal goat serum to minimize unspecific binding of the primary antibody. Sections were heated in 0.1 M citrate buffer (pH 6) for 30 min for heat-induced epitope retrieval and incubated overnight with the monoclonal anti-S100A4 antibody produced in the mouse (Sigma-Aldrich, Vienna, Austria; catalogue number AMAb90599, Prestige Antibodies®, clone CL0240, 1:3000 dilution). Antibodies of this series were developed and validated by the Human Protein Atlas project (www.proteinatlas.org), an international program for systematic exploration of the human proteome using (monospecific) antibody-based proteomics. The antibody binds to an epitope located within the peptide sequence “CNEFFEGFPD” present in the C-terminal region of canine S100A4 identically encoded by all three validated transcript variants (NCBI’s accession numbers: NM_001003161.3, NM_001363554.1 and NM_001362597.3). Polyclonal anti-mouse IgG (H + L) antibody produced in the goat (Immunologic, Duiven, Netherlands; Poly-HRP anti-mouse IgG (ready-to-use)) was applied as secondary antibody for 30 min at room temperature. Staining with diaminobenzidine (DAB) produced a brownish HRP/DAB-complex. For nuclear counterstaining haematoxylin was used. In general, the complete tumour area was subjected to qIHC scoring performed as follows. The image of the stained tissue was digitized using a slide scanner (Aperio Scanscope, San Diego, CA, USA) at 20-fold magnification and converted to a tagged image file format with a resolution of 1 μm/pixel. The percentage of tissue area positively stained with DAB was calculated in relation to the whole tissue area using a self-made script (File S2) run under the open-source platform for biological-image analysis FIJI (https://imagej.net/Fiji). In brief, the tumour area was selected and its area measured. Colour deconvolution, i.e. unmixing using the predefined colour vectors for DAB and haematoxylin, separated the colours of the target signal and the nuclei. A bilateral filter was applied to the image showing the DAB signal for removing noise. White intensity values of more than 200 were considered as background. Applying a particle size of at least 15μm^2^ reduced granular-staining artefacts.

### Statistical analysis

Normality of transcript expression values of stably expressed genes (SEGs) was examined by the Kolmogorov-Smirnov normality test. If the null hypothesis of the test (that the distribution of data is normal) could not be rejected, outlying values were identified based on the Grubb’s test run using the R package “outliers” (https://cran.r-project.org/web/packages/outliers/index.html). Strength and direction of the monotonic relationship between two given variables was evaluated by Spearman’s *ρ* recommended for use with data that are skewed or have outliers. The strength of a linear relationship between two variables was quantified with Pearson’s *r*. If not otherwise indicated, statistical analysis was performed in the GraphPad Prism demo version 5 for Windows (GraphPad Software, Inc., La Jolla, CA, USA).

Logit transformation, one of two most common variance-stabilizing transformations, was applied to the score of qIHC. Being a proportion, the score is constrained to lie between 0 and 1, and its possible variance hence depends on its mean value (more spread is possible at a mean proportion of 0.5 than at proportions of zero and one). Logit transformation calculated according to logit(*p*) = log_e_(*p*/(1-*p*)), where *p* is the proportion value of qIHC, expands the ends of the scale allowing variances at different mean values to be more naturally compared.

Observing that the normalized *S100A4* transcript levels varied almost over two orders of magnitude across the cohort of osteosarcomas, we decided to seek correlations between the transformed qIHC scores and the logarithm of the normalized mRNA abundances. Both gene expression levels were thus subjected to a logarithmic-style transformation.

## Results

### Unsupervised hierarchical clustering of the mRNA-Seq data

**Fig. 1:**
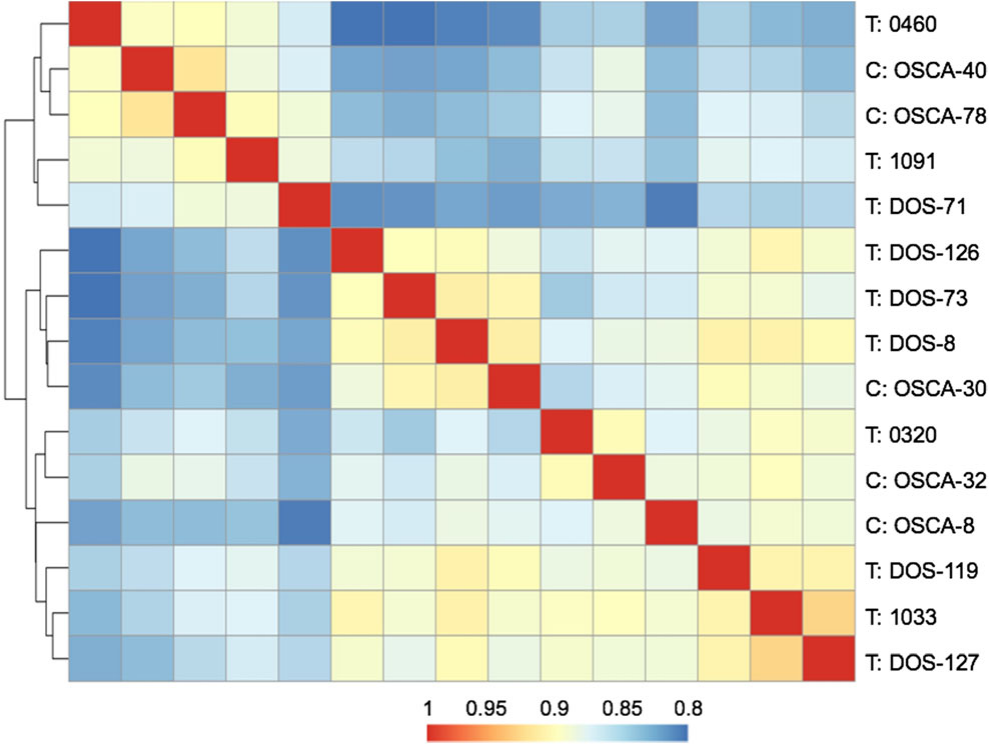
Canine osteosarcoma samples segregate into three main clusters according to unsupervised hierarchical cluster analysis of mRNA-Seq data. The decreasing level of expression correlation is illustrated by red to blue colour (Spearman’s *ρ* of 1 to 0.8, respectively). Samples: set 1. T: osteosarcoma tissue, C: primary cell culture of osteosarcoma

The poly(A) fraction of the transcriptome was extracted as template for mRNA-Seq-based selection of exon sequences showing uniform expression across canine osteosarcomas. Hierarchical cluster analysis of mRNA-Seq expression data visualized by a heatmap (Fig. 1) stratified the ten osteosarcoma tissues and five osteosarcoma cell lines analysed (set 1: Table S1) into three main clusters represented by five, four and six tissues or cells (top, middle and bottom clusters, respectively). The same main clusters were obtained when the mRNA-Seq data was aligned to the genome of the (Australian) dingo (*Canis lupus dingo*) instead of the dog (Fig. S2).

Noticeable, the osteosarcoma tissue DOS-8 and the cell line derived from it, OSCA-8, did not cluster together (Fig. 1). This difference in transcriptional programs might be due to changes in epigenetic marks that would likely be more sensitive to culture conditions than DNA sequences [14], to some loss of heterozygosity coming with increasing passage number of cell culture (Data S1) and/or the absence of stromal cell-specific signature from the cell line’s expression profile. Likewise, it can be caused by clonal expansion of aneuploidy [15], i.e. genomic heterogeneity in the cell line’s tumour of origin.

### Ranking mRNA-Seq data based on stability of expression

**Fig. 2:**
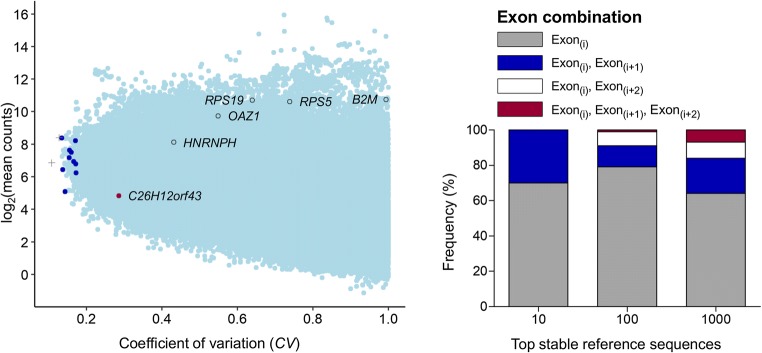
Stability of single exons or pairs of neighbour exons based on *CV* in mRNA-Seq data of canine osteosarcoma (sample set 1). *CV* values were plotted against mRNA abundance representing *log*_*2*_-transformed transcripts per kilobase million. Single exons and pairs of neighbour exons used for RT-qPCR validation were depicted by grey plus signs and filled dots of light blue colour, respectively. Gene symbols highlight SEGs used in previous RT-qPCR studies of canine osteosarcoma derived from educated guessing (*B2M*, *RPS5*, *RPS19*, *HNRNPH*), from interspecies expression stability assessment of transcriptome data (*OAZ1)* or by array-based comparative genomic hybridization for the context (*C26H12orf43*) (references in Table S5). Their most stable two consecutive exons are exemplarily depicted. Common traditional normalizers such as *GAPDH* and *HPRT1* even exceeded the threshold of *CV* ≤ 1 (data not shown). Note that the Ensembl Genome Browser used for read mapping does not list *OASL* and *C26H12orf43* as separate genes in contrast to the NCBI database

Single or neighbouring exons determined by mRNA-Seq profiling were ranked for stability based on their *CV* of expression. A lower value indicated a more stable expression across the condition. We limited the analysis to a *CV* of ≤ 1 and the following four exon combinations: single exons (*i*), pairs of direct exon-neighbours (*i*, *i* + 1), pairs of the exon *i* together with the exon skipping the one thereafter (*i*, *i* + 2) and three consecutive exon neighbours (*i*, *i* + 1, *i* + 2). The fraction of neighbour exons accounted for 30, 20 and 29% of the top 10, 100 and 1000 sequences, respectively (Fig. 2). Next, we compared the transcriptional stability of several normalizers that were formerly recommended for use in canine osteosarcoma without transcriptome-wide expression profiling for this context such as *OAZ1* [16] or *B2M, HNRNPH*, *RPS5* and *RPS19* [17] (Table S5). This set consisted of classical normalizers that were derived from educated guesswork (e.g. *B2M*) and reference genes showing a more universal expression uniformity across mammalian cells and tissues, e.g. *OAZ1* [18]. In a single case, gene selection was guided by genomic data produced for the pathophysiological study condition (*OASL*, formerly termed *C26H12orf43*, [19]). We obtained considerably lower *CV* values for our novel sequences (0.14–0.17 versus 0.29–0.99) indicating a clearly higher stability of transcript expression (Fig. 2). Common classical SEGs, such as *GAPDH* and *HPRT1*, even exceeded the threshold of *CV* ≤ 1 (details in Data S2)*.*

**Fig. 3:**
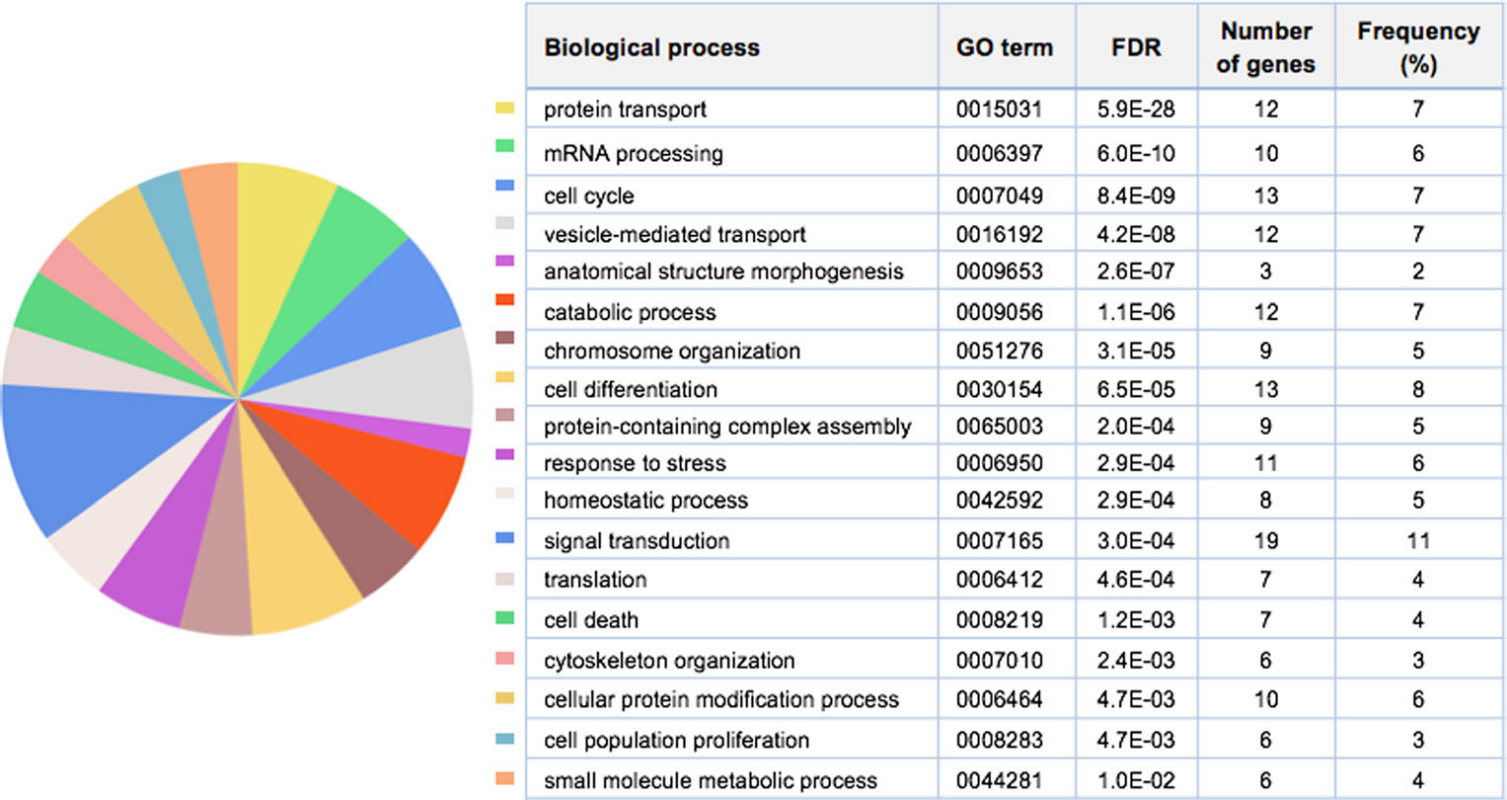
The hundred most stable genes are enriched in key biological processes. Ranking of genes and biological processes is based on *CV* and false discovery rate (FDR), respectively. *P* values were obtained by Fisher’s exact test and corrected by the Benjamini-Hochberg post hoc method. Gene number: number of genes from the input list assigned to a certain process category. Frequency: number of genes of the input list annotated to a particular GO term divided by its total gene number (illustrated by the size of sectors in the pie chart)

To identify overrepresented GO categories among the most stable genes, we composed a list that exemplarily comprised a number of 100 genes with the highest stability of transcript expression. Each of the genes was represented by either a single exon or a combination of neighbouring exons ranked top for expression stability. Pathway enrichment analysis performed for this gene set and calibrated by the total number of genes with detectable expression delivered the by far best statistical support for the biological process of protein transport followed by the GO term of mRNA processing (false-discovery rates of 5.9E-28 and 6.0E-10, respectively; Fig. 3). Analysing the gene panel independently at the GeneMANIA database identified “mRNA processing” as the most enriched biological process (FDR of 3.2E-13).

### RT-qPCR-based validation of expression stability for the exonic sequences

**Fig. 4:**
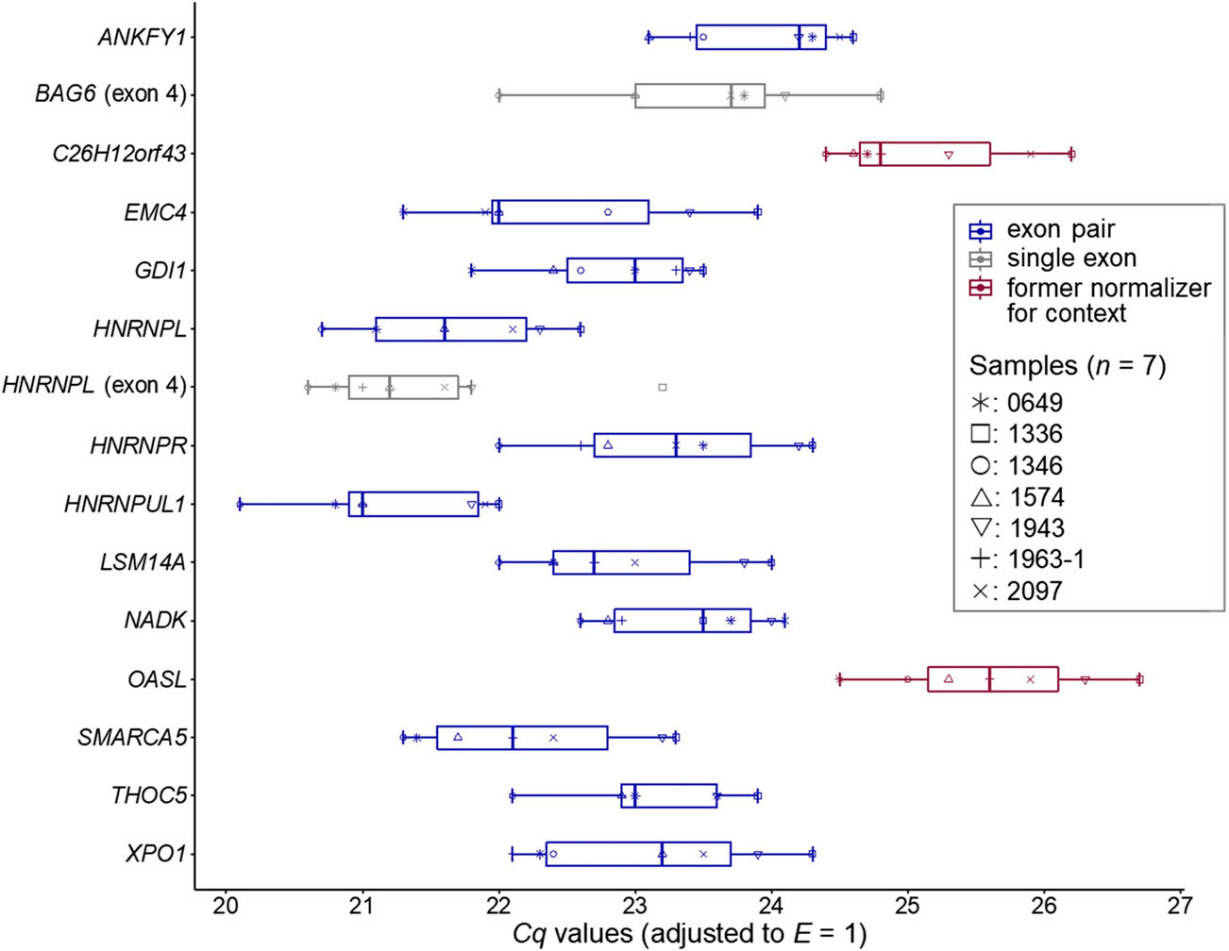
Tukey box plot depicting the abundance range of single or neighbouring exons for canine osteosarcoma tissues (sample set 2). Boxes represent the lower and upper quartiles centered on the median. Whiskers indicate the Tukey confidence intervals. Blue and grey: exon pair or single exon identified by mRNA-Seq analysis, respectively (this study), red: RT-qPCR normalizers identified by array-based comparative genomic hybridization for the biological context [19]. Numbering of the respective exons is provided in Table S3. The R code used for generating the plot is provided as File S3

Eleven neighbour exons and two single exons showing highest expression stability according to mRNA-Seq analysis were validated by RT-qPCR (Fig. 4) using a second set of osteosarcomas (set 2, *n* = 7, Table S2). To avoid confounding by deregulation at the level of circRNAs and/or rRNAs, validation started from the same type of template, i.e*.* the poly(A) fraction of the transcriptome. For comparison, we included *C26H12orf43* that was supported by genomic data produced for the experimental condition (formerly termed *LOC611555* [19]). We regarded the delayed (efficiency-adjusted) *Cq* value obtained for sample #1336 in the assay against the single *HNRNPL* exon a possible case of an outlying late amplification (Grubb’s test: *p* = 0.07) that might be caused by a polymorphic primer-binding site [20] or a structured amplicon [21]. *CV* values of down to 2.5–4.1% calculated for the novel sequences indicated an exceptional high uniformity of expression (Data S3). To identify suitable gene combinations for composing a *NF*, exons were evaluated for expression stability by the four common statistical algorithms geNorm, BestKeeper, NormFinder and the comparative *ΔCq* method (Data S4). Based on the pairwise-variation cut-off of *V*_*n/*_*V*_*n + 1*_ < 0.15 recommended by geNorm for two consecutive normalization factors, *NF*_*n*_ and *NF*_*n + 1*_, we concluded that two genes were sufficient for normalization (*V*_*2/3*_ = 0.07, Fig. S3). This minimum gene number was adopted to compose the *NF*s for the other statistical tools. The rank lists calculated by the four statistical tools were subjected to rank aggregation analysis using the RankAggreg package (Data S4). This pre-screening identified the consecutive exon pairs of the genes *LSM14A*, *THOC5* and *HNRNPL* as most stable. Next, we analysed how much the genes and their pairwise *NF* combinations impacted the quantitative relationship between the transcript abundance of *S100A4* and the expression of the translated protein expressed as qIHC score. This experimental “readout” identified the *NF* composed of *THOC5* and *HNRNPL* as the most appropriate normalization choice (Data S5). While sharing molecular functions like RNA and protein binding (GO classifications 0003723 and 0005515, respectively), the two genes participate in different biological processes (*THOC5*: mRNA export from nucleus (GO:0006406), *HNRNPL*: regulation of alternative mRNA splicing, via spliceosome (GO:0000381)) and are poorly co-expressed according to the gene co-expression database COXPRESdb. Hence, they represent an appropriate *NF* combination to normalize RT-qPCR data of canine osteosarcoma at the template level of poly(A) RNA.

*THOC5* and *HNRNPL* are also choices of normalization for studies that involve normal osteoblasts as indicated by similar *Cq* values (data not shown; template type: poly(A) RNA).

### Demonstrations of improved RT-qPCR data normalization: pairwise co-expression of mRNA variants of *S100A4* and the gene’s relationship between transcript and protein expression levels

**Fig. 5:**
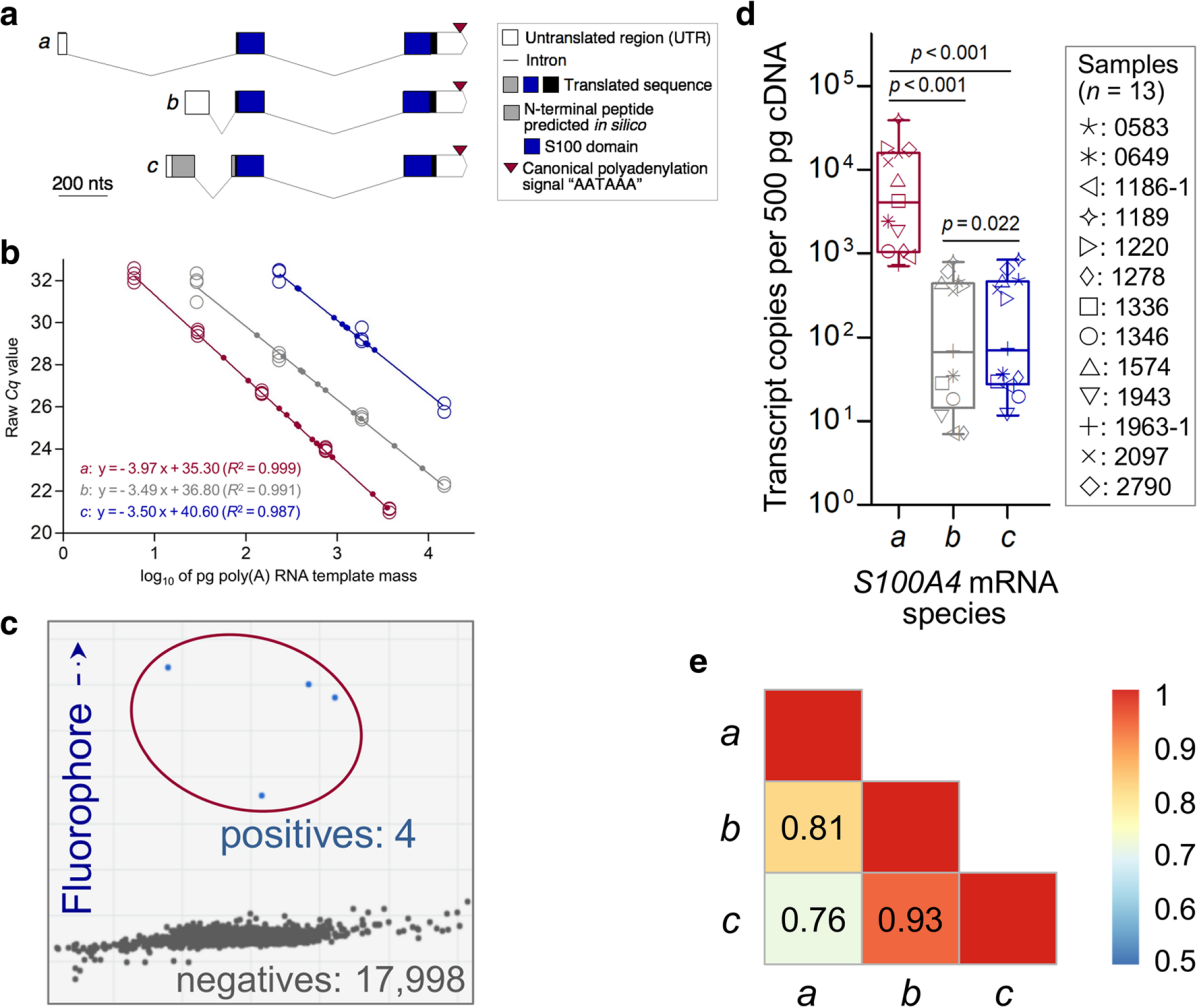
*S100A4* mRNAs in canine osteosarcoma: structure, copy numbers and pairwise correlation of variants. **a** Exon-intron structure of validated *S100A4* transcripts *a*, *b* and *c* (GenBank IDs: NM_001003161.3,NM_001363554.1 and NM_001362597.2, respectively). The length of the 3′ UTR and the common distance between the canonical poly(A) signal AAUAAA and the poly(A) stretch (~ 21 nucleotides) were confirmed by Sanger sequencing of amplicons produced by Rapid Amplification of 3’ cDNA Ends (consensus primers: GenBank accession number MK138547, primers against transcript variant *c*: MK584633). A putative proximal, non-canonical signal for alternative polyadenylation predicted in silico (Table S7) is not presented. We note that the N-terminal peptide extension predicted for the dog/dingo (GenBank accessions NP_001349526.2 and XP_025284715, respectively) or feline species (*Acinonyx jubatus*: XP_014941010.1, *Panthera pardus*: XP_019287133.1) still awaits experimental validation. **b** Dynamic ranges of qPCRs applied to quantify the *S100A4* mRNAs. **c** dPCR plot of a sample that did not reach the limit of quantification in the respective qPCR assay (sample: #1186–1, assay against minor variant *b*). **d** Copy numbers of *S100A4* mRNAs normalized by the novel *NF* (geometric mean of two consecutive exons of *HNRNPL* and *THOC5*) and assessed for significance by the nonparametric Wilcoxon Signed Rank Test for paired data. **e** Pairwise co-expressions across variants evaluated by Spearman’s rank correlation coefficient (blue to red: fair to perfect correlation)

As a first demonstration of technical noise reduction in transcript quantification achieved by our improved normalization strategy, we evaluated the extent of pairwise co-expressions among the *S100A4* transcripts *a*, *b* and *c* across canine osteosarcomas (Fig. 5a). Proving their expression in the study context required a decision upon the template type for RT-qPCR, total cellular RNA or its fraction enriched for poly(A) RNA. On total RNA templates, we found a consistent expression of the major transcript species *a* across the 13 samples of the cohort (range of *Cq* values for quantification: 25.1 to 34.1, Data S6). However, profiling the less-abundant variants *b* and *c* on the same templates resulted in single-negative or even double-negative amplification events (variant *b*: samples #1278 and #1943; variants *b* and *c*: #0649 and #1346; Data S6). Therefore, we considered to reduce the high level of nonspecific background RNA that was previously shown to negatively affect the conversion efficiency of the RT enzyme, particularly in the case of a rare target [22]. We argued that switching to a poly(A) RNA template type would diminish this background effect, and thus, could reveal false-negative detection. Indeed, on the less dense template, the minor *S100A4* mRNA variants *b* and *c* were consistently detected (Fig. 5b, Data S6, *n* = 13). Remarkably, the more sensitive detection of the minor variants was achieved, although only about one-ninth of the mRNA amount was used as template, a calculation that assumed a 3 to 7% proportion of the mRNA fraction in total cellular RNA [23]. Samples #1186–1 and #1278 did not reach the limit of quantification in the assays against *S100A4* transcripts *b* and *c*. Therefore, their expression was alternatively determined by dPCR (Data S7). Partitioning into many independent PCR sub-reactions applied by dPCR provides accuracy even for rare targets [24]. Copy number counting based on dPCR also confirmed that transcript *a* represented the by far most abundant species of *S100A4* (Fig. 5d, *p* < 0.001). The minor transcript variants *b* and *c* individually contributed just 2.5% to the total amount of *S100A4* transcripts (Data S6). Combining qPCR and dPCR data and normalizing with the novel *NF* revealed strong or even extraordinarily strong pairwise co-expression across the *S100A4* transcript variants (*a* with either *b* or *c*: *ρ* = 0.81/ 0.76, *r* = 0.92/ 0.90; *b* with *c*: *ρ* = 0.93, *r* = 0.99; Fig. 5e and data not shown).

**Fig. 6:**
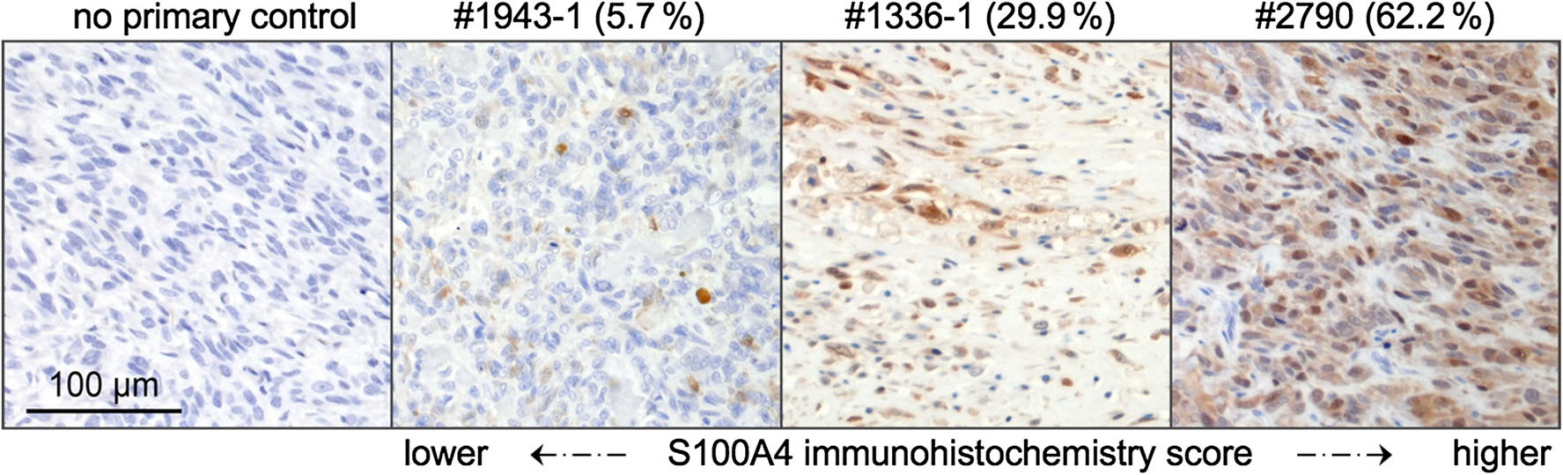
Automatic quantification of S100A4 distribution in paraffin-embedded sections of canine osteosarcoma by qIHC. Representative samples with high, moderate and low frequencies of positive areas (areas with brown staining) are depicted from right to left. Leftmost: negative control without primary antibody

As a second demonstration of the technical-noise reduction in transcript measurement, we sought to uncover the association between the overall abundance of *S100A4* transcripts and the amount of the S100A4 protein expressed as spatially resolved quantitative distribution across a thin tumour section (qIHC score, exemplified in Fig. 6)*.* Since the level of *S100A4* transcript expression was high enough, we could favour total RNA as the cheaper and easier to isolate, hence more common template over poly(A) RNA (Fig. S4). We converted the relative *S100A4* transcript expression values determined by RT-qPCR into copy numbers using a calibrator sample measured by qPCR as well as dPCR (Data S7).

**Fig. 7:**
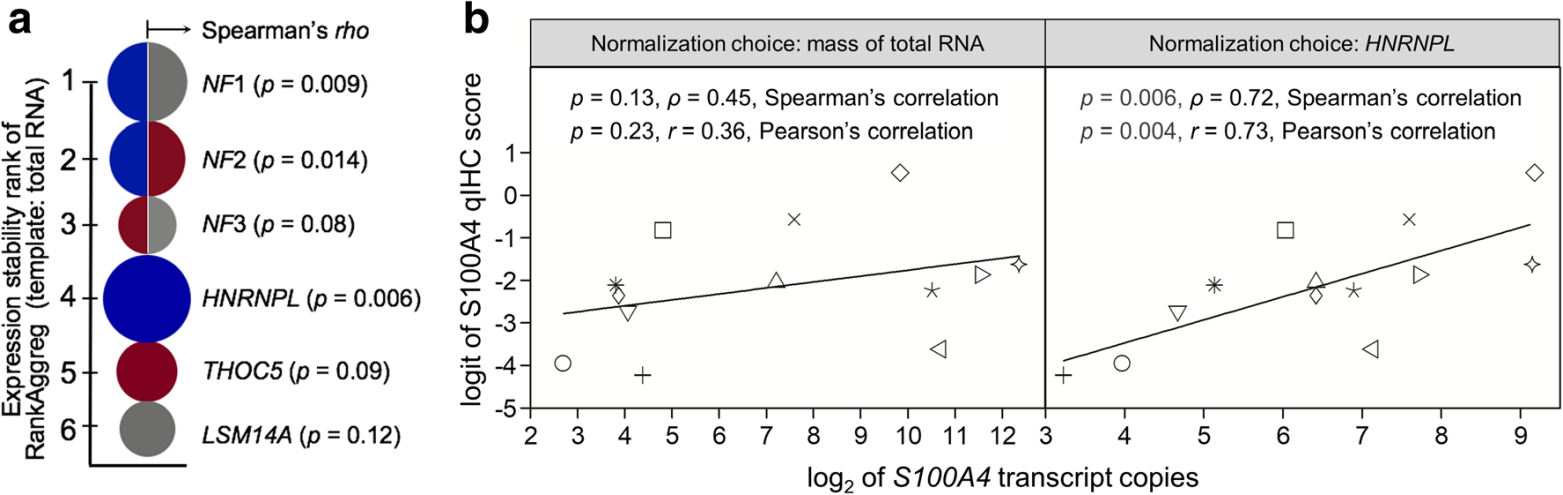
Noise reduction in transcript normalization uncovered association between mRNA and protein expressions of *S100A4* in canine osteosarcoma. **a** “Pearl” diagram presenting the stability of the three reference genes (depicted by colour) most stably expressed at the template level of poly(A) RNA in comparison to their two-gene combinations (*NF*1 to *NF*3) (depicted by bicolour circles). The best normalization choice for total RNA templates was determined by rank aggregation. The noise reduction of transcript measurement resulting from better normalization of target gene expression is illustrated by the increased circle radius that is proportional to the Spearman’s rank correlation coefficient (*ρ*) obtained for the linear association between mRNA abundance of *S100A4* and proportion of tumour area positive for its protein (Fig. 7b). **b** Copy number of *S100A4* mRNA was normalized by either the input amount of template in cDNA synthesis (left) or the best normalizer identified by using expression correlation analysis as the experimental “readout” (Fig. 7a, Data S5). *ρ*: Spearman’s rank correlation coefficient; *r*: Pearson’s correlation coefficient; *p*: significance value

As a starting point, we normalized the abundance of *S100A4* mRNA with the mass of the RT template. However, we were unable to detect a relationship between the two gene expression levels of *S100A4* using this approach (*p =* 0.13, *ρ =* 0.45, Spearman’s correlation; *p =* 0.23, *r* = 0.36, Pearson’s correlation) (Fig. 7b left, Data S8). This might be caused by technical variation between samples and measurements (extraction, quality (purity and integrity) and concentration assessment of RNA, cDNA sample loading as well as different efficiencies of cDNA synthesis and subsequent exponential amplification). Alternatively, we assessed the outcome of normalization with one or more stably expressed internal control genes. As candidates, we selected the three normalizers *HNRNPL, THOC5* and *LSM14A* that exhibited the most stable expression in poly(A) RNA templates (Data S4) and their pairwise *NF* combinations. Correlation analysis between the expression intensity of *S100A4* at the mRNA and protein levels was used as the experimental “readout” or “post-control” for their re-evaluation. This was required to compensate for attributing the same weight to every stability algorithm, a practical option without any biological meaning that is arguable because some of the analytical approaches applied here for stability assessment include redundant information [25], as well as to compensate for changing the type of RNA template and the composition of the RNA cohort. We found that the choice of normalization strongly impacted the uncovering of relationship between expression intensity of *S100A4* at the mRNA and protein levels. Three of the six normalization choices resulted in a significant or even highly significant strong positive correlation between the expression levels (*HNRNPL*: *p* = 0.006, Spearman’s *ρ* = 0.72; *NF*1: *p* = 0.009, *ρ* = 0.69; *NF*2: *p* = 0.014, *ρ* = 0.66; Fig. 7a and Data S5). In contrast, we could not establish a relationship by normalizing with the other three choices (*NF*3: *p* = 0.078, *ρ* = 0.51; *THOC5*: *p* = 0.086, *ρ* = 0.50; *LSM14A: p* = 0.117, *ρ* = 0.46). We note that the corresponding Pearson’s coefficients were very similar (Data S5). We also note that the aggregate rank order of the gene stability algorithms was partly different from the list obtained by our experimental “readout” (Fig. 7a). Based on the latter, we regarded *HNRNPL* as the best RT-qPCR normalizer for canine osteosarcoma at the level of total RNA templates (Spearman’s correlation: *p =* 0.006, *ρ* = 0.72; Pearson’s correlation: *p =* 0.004, *r* = 0.73; Fig. 7a and b right, Data S5). Expectedly, the logit transformation of qIHC scores improved Pearson’s correlation (poly(A) RNA templates: *r* = 0.47; *p* = 0.108 versus *r* = 0.65 (*p* = 0.016); total RNA templates: *r* = 0.64 (*p* = 0.019) versus *r* = 0.73 (*p =* 0.004)).

Out of the three mRNAs of canine *S100A4*, transcript variant *a* showed the highest strength of association with the qIHC score of the translated protein (Fig. S5), a finding that is not surprising given its 95% contribution to the overall abundance of *S100A4* transcripts (Fig. 5d). Also note that for the large tumour of sample #0649, we determined two qIHC scores (Section 1: 3.1%; Section 2: 10.0%) and included only the score that yielded a better linear regression between the expression levels.

## Discussion

**Fig. 8:**
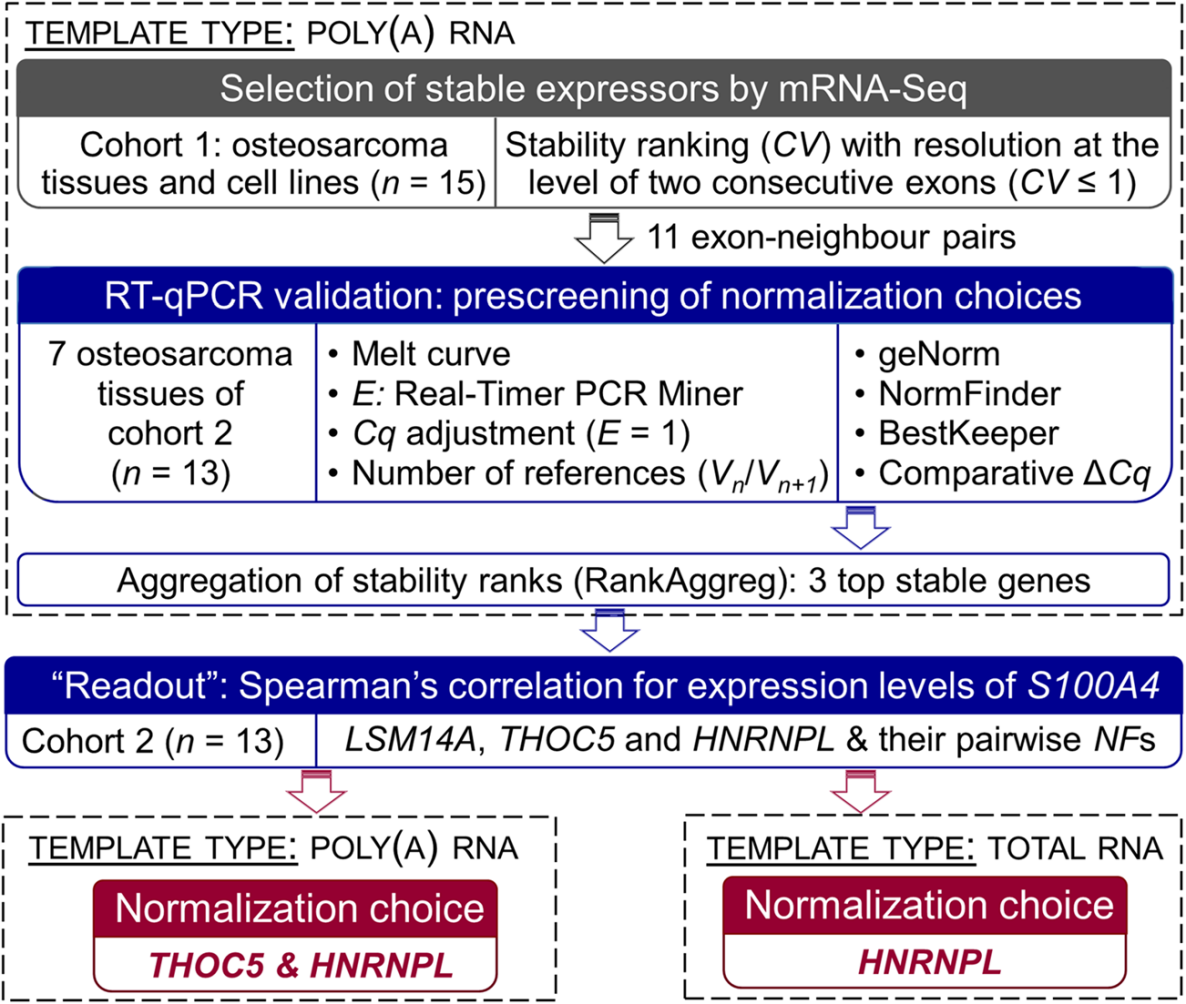
Methodology to select stable sequences for accurate normalization of RT-qPCR data in the context of canine osteosarcoma. The grey section shows the identification of neighbouring exon pairs with stable expression based on their *CV* in poly(A)-transcriptome sequencing by mRNA-Seq (grey box). To validate their expression stability by RT-qPCR, the same template type as of mRNA-Seq, i.e. poly(A) RNA was targeted (upper dashed box), and multiple statistical algorithms were applied (blue box). The exon pairs of *HNRNPL, THOC5* and *LSM14A* (Fig. 7a) were most stably expressed in poly(A) RNA templates according to rank aggregation (RankAggreg algorithm [48]). Together with their pairwise combinations (*NF*s) they were re-evaluated on the template level of total RNA. Strength of relationship between the expression intensity of *S100A4* at the mRNA and protein levels was used as the experimental “readout” for re-evaluation. We introduced this “post-control” to enable compensation for attributing the same weight to every stability algorithm, a practical option without any biological meaning that is arguable because some of the analytical approaches applied here for stability assessment include redundant information [25], as well as to compensate for changing the type of RNA template (right dashed box) and the composition of the RNA cohort. *HNRNPL*, a gene that exhibits a predominantly consistent expression across a wide range of human cancers [35], alone or together with *THOC5* was proposed by the workflow as normalization choice for total RNA or poly(A) RNA templates isolated from osteosarcomas (red boxes)

The correlation between mRNA and protein abundances depends on various biological and technical factors. Here we reduced the error of transcript measurement, exemplarily demonstrated for the target gene *S100A4* and dog osteosarcoma, a comparative oncology model. This facilitated uncovering association between the gene’s transcript expression and the abundance of the resulting protein measured in the context of tissue morphology (qIHC score). Two crucial parameters minimized the technical artefact in RT-qPCR data normalization required to accurately quantify the expression of a target mRNA, (*i*) selecting SEGs from mRNA-Seq data that were produced for the pathophysiological study condition and mined at exon-level resolution and (*ii*) using poly(A) RNA also as the template type for their confirmation by RT-qPCR (Fig. 8).

Expression profiling by next-generation sequencing offers the option to identify SEGs at exon resolution [26, 27]. This level of sequence resolution reduces interference from putative alternative splicing events (reviewed in [28]) that are hallmarks of cancer and determinants for its progression [29]. In this study, we asked whether an exon-neighbour pair would exhibit high expression stability irrespective of the heterogeneity and mutational burden reported for tumour phenotypes [30]. The splice junction between the two exons would facilitate a robust, intron-spanning qPCR assay design. Indeed, we found 11 genes that contributed pairs of consecutive exons to the top 100 most stable expressors (Data S2). Irrespectively of the heterogeneous nature of the osteosarcoma tumour model studied, our SEGs selected by mRNA-Seq with exon-level resolution showed a rather narrow range of expression variation across osteosarcoma tissues (*CV*s: 10.4 to 17.1%, *n* = 10, Table S8). This stability was similar to that reported for relatively homogeneous samples comprised of cells and tissue of a non-pathological condition (*CV*s of ~ 15% [31] and 8 to 18% [27]). More heterogeneous material, e.g. various cancer types can produce similar expression stabilities (~ 10% for normal and malignant prostate tissue and > 13% or > 16% for two types of lung cancer [32]), but also, a considerably higher variation in the case of neuroblastoma, where high cellular heterogeneity is a hallmark (~ 30%; [31]). Notably, expression variation across the 13 exonic normalizer sequences was found to be lower when quantified by RT-qPCR (2.5 to 4.1%, Data S3) compared to mRNA-Seq measurement (Data S2). We note that each dataset was produced from an independent cohort of osteosarcoma tissues randomly sampled (*n* = 7 and 10, respectively). Therefore, the discrepancy should be attributed to the higher technical noise putatively associated with traditional mRNA-Seq protocols including the one applied in our study. Such protocols can skew quantification due to inherent errors of PCR amplification required to obtain sufficient material for quantification. More recently, more accurate gene expression resulted from introduction of unique molecular indices into library construction [33].

Irrespectively of the highly heterogeneous nature of our tumour condition, just two reference sequences were sufficient for reliable normalization of RT-qPCR data according to expression stability assessment using the statistical algorithm geNorm. Typically, such remarkably high stability is reported only for less complex physiological contexts (Table S6). In other terms, our stable exons varied only little in expression according to the RT-qPCR data (osteosarcoma tissues of set 2, *n* = 7). For example, the genes that composed the final *NF, THOC5* and *HNRNPL*, showed a 3.6- and 3.7-fold difference between minimum and maximum expression. Slightly less variation of expression (~ 2-fold) was determined earlier at our facility when selecting SEGs for the intestine of laboratory mice [34]. To enable optimal precision, both studies used cDNA replication and a qPCR assay setup automated by a liquid handling system. We attributed the slightly higher fluctuation in our tumour context to the prolonged time required for processing an osteosarcoma tissue compared to intestine tissue derived from a laboratory animal model such as the mouse and to the general intrinsic heterogeneity and genetic instability of tumours. We also note that *HNRNPL* was recently declared as consensus reference gene for a range of human cancer types based on large-scale expression datasets [35].

Another key requirement of our transcript measurement approach was the consistent use of poly(A) RNA as template type across selection and verification of SEGs performed by mRNA-Seq and RT-qPCR analyses, respectively. This avoided interference from altered proportions of mRNA to rRNA [36]. The two transcriptome fractions are synthesized by different RNA polymerases, polymerases I and II, and show different proportions in various physiological or disease contexts related to proliferation and cellular biogenesis. For example, rRNA expression can be affected by biological factors such as viral infections [37], drugs [38], cancer [39] and exercise [40]. The use of poly(A) RNA instead of total RNA as the template in RT-qPCR considers the generally assumed fact that tumour cells overexpress rRNA species and that the processing of the polycistronic 45S rRNA transcript into the mature 28S, 18S and 5.8S rRNA species is a multistep process subject to many potential modes of regulation [41]. The quantitative dominance of rRNAs species in total cellular RNA can cause altered rRNA-to-mRNA ratios across tumour samples of different grading and staging and can finally impair identification of SEGs [42]. Our study context, osteosarcoma, belongs to the tumour types where high proliferation is accompanied by a remarkable reduction of the number of 45S rDNA repeats [43] that give rise to the three most abundant components of cellular RNA 28S, 5.8S and 18S. The species can be individually modulated and be aberrantly expressed between tumour and normal tissues [41] or even across specimens of the same type of tumour [36]. In addition, using poly(A) templates across SEG selection and validation avoids confounding from non-polyadenylated circRNAs that are derived by back-splicing from pre-mRNAs [44] and are practically absent from mRNA-Seq data. Except of the unique back-splice junction sequence, non-polyadenylated circRNAs are indistinguishable from the mRNA sequence and thus can distort the matching between mRNA-Seq and RT-qPCR data if the latter was produced on total RNA templates [45]. Taken together, we regarded template-type similarity as a key and obvious requirement to select and verify SEGs that should not be neglected. Irrespectively of the arguments listed above, this important point was commonly ignored by earlier studies identifying SEGs from mRNA-Seq data (Table S6).

The power of our novel normalization choice was exemplarily demonstrated by quantifying the abundance of a chosen target transcript (*S100A4* mRNA) using RT-qPCR. We found that the novel normalizer, *HNRNPL*, produced a shift from no to high significance for the correlation between mRNA abundance and the qIHC score of the tumour marker S100A4 compared to normalization by RNA input mass (Fig. 7b right). The fact that the qIHC score represents a proportion, and thus is constrained to lie between 0 and 1, means that care must be taken in its statistical analysis, because the possible qIHC variance depends on its mean level. In our study, we used a logit transformation to account for this structure, allowing variances from different mean qIHC scores to be more naturally compared. This was essential to uncovering the association between *S100A4* transcript abundance and the proportion of S100A4-positive tumour area. However, variance-stabilizing transformations have been previously neglected when regressing a target mRNA with the qIHC proportion score of the corresponding protein. The association between the two gene expression levels of S100A4 was uncovered even though the extracellular localization of S100A4 is increased in the state of cancer [46] and transcript counts and qIHC scores originated from different sections of a tumour tissue, frozen or formalin-fixed, paraffin-embedded (FFPE) sections, respectively. Future investigation should clarify whether replicated qIHC measurement would provide potential to further improve quality of correlation between mRNA and protein expression. For PCR-based DNA amplification, technical replication represents an intrinsic requirement due to its exponential nature. Technical replication in qPCR-based quantification, for example, allows exclusion of bad data points, e.g. outlying *Cq* values in case of more than two replicates. Currently, replicate measurement of a sample’s qIHC score is not common. This might be explained by the attempt to avoid resource allocation conflicts that occur when competing needs require the use of scarce or valuable tumour material, the linear character of the immunostaining signal or the higher experimental costs. Although true technical replication is nearly impossible in qIHC analysis due to the spatial heterogeneity of a tumour, future investigation should issue the potential of replicate qIHC scoring for fine-tuning linear regression between mRNA counts and qIHC scores of a particular gene. For example, the coefficient of determination (*r*^2^) could be used as indicator of how well the experimental data fit the regression line. The maximum *r*^2^ could point to paired cryosections and FFPE tissue samples derived from a certain tumour sample that fit best with the regression line and thus could assist in the removal of the less-matching qIHC replicate. A related approach is commonly used to determine the optimal fluorescence threshold to generate the best fitting curve from serial standard dilutions in qPCR [47].

## Electronic supplementary material

ESM 1 (XLSX 28 kb)

ESM 2 (XLSX 12 kb)

ESM 3 (XLSX 14 kb)

ESM 4 (XLSX 12 kb)

ESM 5 (XLSX 11 kb)

ESM 6 (XLSX 15 kb)

ESM 7 (DOCX 18 kb)

ESM 8 (XLSX 30.3 kb)

ESM 9 (DOCX 384 kb)

ESM 10 (DOCX 2366 kb)

ESM 11 (DOCX 139 kb)

ESM 12 (DOCX 702 kb)

ESM 13 (DOCX 429 kb)

ESM 14 (DOCX 83 kb)

ESM 15 (DOCX 114 kb)

ESM 16 (R 1 kb)

ESM 17 (TXT 5 kb)

ESM 18 (R 2 kb)

## Data Availability

The mRNA-Seq data reported in this article has been deposited in NCBI’s Gene Expression Omnibus (accession GSE147550).RNA-seq data of samples DOS-8, DOS-73, OSCA-8, OSCA-78 were submitted earlier to the Gene Expression Omnibus (https://urldefense.proofpoint.com/v2/url?u=http-3A__www.ncbi.nlm.nih.gov_geo&d=DwIGaQ&c=vh6FgFnduejNhPPD0fl_yRaSfZy8CWbWnIf4XJhSqx8&r=SP79iS6nw5Lwa7x8SWJpMZXA77zhJY0DVjp7Ka6Qhk8&m=icC698z5kLpXNKs5Wpu3jMEC0Uwy8TRj2oo7QL4L1Qk&s=-Bf_72bI-2jwtpc9bQk4nSvxojsBV8oCKTuKT9cOsWY&e= ; accession number GSE95185). For further details and references see Supplementary Materials and Methods available online.

## Abbreviations

circRNAs: Circular RNAs
*Cq*: Cycle of quantification
*CV*: Coefficient of variation
dPCR: Digital PCR
*E*: Amplification efficiency of qPCR
GO: Gene ontology
NCBI: National Center for Biotechnology Information
*NF*: Normalization factor
qIHC: Quantitative immunohistochemistry
mRNA-Seq: Poly(A) RNA sequencing
RT-qPCR: Reverse-transcription quantitative PCR
SEG: Stably expressed gene
S100A4: S100 calcium binding protein A4
UTR: Untranslated region
