# Supplementary material for: S100A4 mRNA-protein relationship uncovered by measurement noise reduction
Source: J Mol Med (Berl). 2020 Apr 15;98(5):735–49. doi: 10.1007/s00109-020-01898-8 (PMC7241963; doi:10.1007/s00109-020-01898-8)
Supplement: Supplementary file 7 — (DOCX 18 kb) [file 109_2020_1898_MOESM7_ESM.docx]

**Data S7. Copy number counting of *S100A4* transcript variants by dPCR**

| **Variant *b*** | | | | | | | |
| --- | --- | --- | --- | --- | --- | --- | --- |
| Sample | | | Template volume (µl) | Copies per μl dPCR | Copies per μl cDNA | | Precision  (%) |
| Name | | Template |  |  | Value | Confidence interval |  |
| Osteoblasts* | | poly(A) RNA | 3.0 | 385.8 | 2,315.0 | 2,245.6 – 2,386.6 | 3.1 |
| #1186-1 (1:6) | | poly(A) RNA | 6.0 | 0.4 | 6.6 | 2.8 - 15.9 | 140.0 |
| #1220 | | poly(A) RNA | 5.0 | 111.3 | 400.6 | 379.8 - 422.6 | 5.5 |
| #1278 | | poly(A) RNA | 3.0 | 1.2 | 7.4 | 4.5 - 12.0 | 63.0 |
| H_2_O | | poly(A) RNA | 6.0 | 0.1 | 0.3 | 0.0 – 1.8 | 610.0 |
| **Variant *c*** | | | | | | | |
| Osteoblasts* | poly(A) RNA | | 1.7 | 393.4 | 4,165.0 | 4,036.4 - 4,295.2 | 3.1 |
| #1186-1 | poly(A) RNA | | 6.0 | 8.7 | 26.1 | 21.7 - 31.5 | 20.6 |
| #1278 | poly(A) RNA | | 2.0 | 3.5 | 31.7 | 23.8 - 42.3 | 33.5 |
| H_2_O | poly(A) RNA | | 6.0 | 0.1 | 0.2 | 0.0 – 1.7 | 610.0 |
| Variants *a*, *b* and *c* (consensus assay) | | | | | | | |
| #2097 | | poly(A) RNA | 2.0 | 1,606.1 | 14,454.9 | 14,185.5 - 14,728.5 | 1.9 |
| #2097 | | total RNA | 6.0 | 28.7 | 86.2 | 78.3 - 95.1 | 10.2 |

Precision refers to the number of positive dPCR partitions. The different volumes of cDNA input resulted from the amount of material available. The concentration of the original cDNA was calculated by taking the volume of input cDNA and the volume of dPCR master mix into account.

*Normal canine osteoblasts (catalogue no. Cn406-05; Cell Applications Inc., San Diego, CA, USA) used as calibrator for translation of *Cq* values into copy numbers.
