# Supplementary material for: S100A4 mRNA-protein relationship uncovered by measurement noise reduction
Source: J Mol Med (Berl). 2020 Apr 15;98(5):735–49. doi: 10.1007/s00109-020-01898-8 (PMC7241963; doi:10.1007/s00109-020-01898-8)
Supplement: Supplementary file 9 — (DOCX 384 kb) [file 109_2020_1898_MOESM9_ESM.docx]

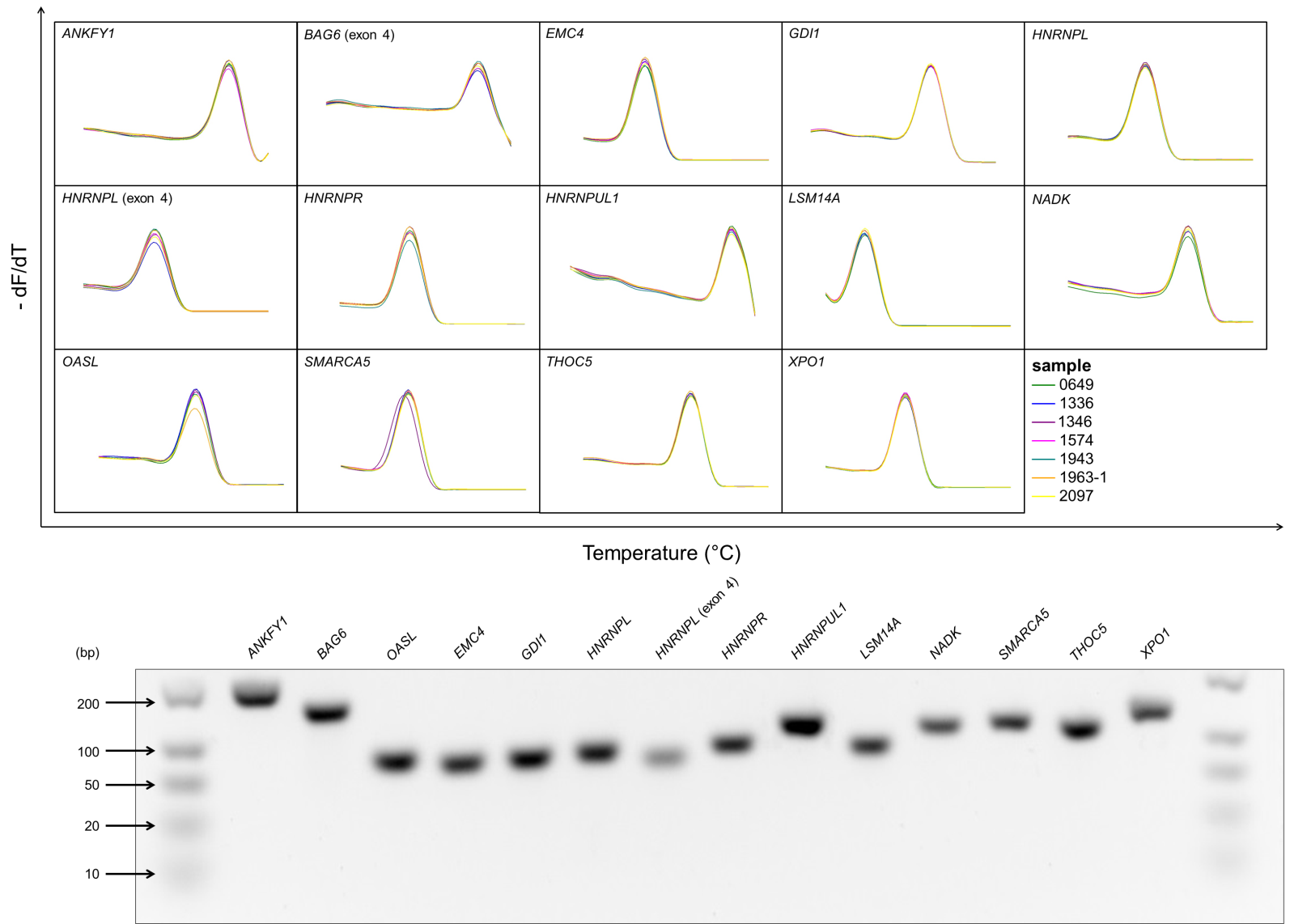


**B**

**A**

**Fig. S1**

**Specificity of qPCR primers was confirmed by melting analysis (A) and 1 % agarose gel electrophoresis (B) of the assay amplicons.** Osteosarcoma samples of set 2 were used for sequence validation.
