# Supplementary material for: S100A4 mRNA-protein relationship uncovered by measurement noise reduction
Source: J Mol Med (Berl). 2020 Apr 15;98(5):735–49. doi: 10.1007/s00109-020-01898-8 (PMC7241963; doi:10.1007/s00109-020-01898-8)
Supplement: Supplementary file 10 — (DOCX 2366 kb) [file 109_2020_1898_MOESM10_ESM.docx]

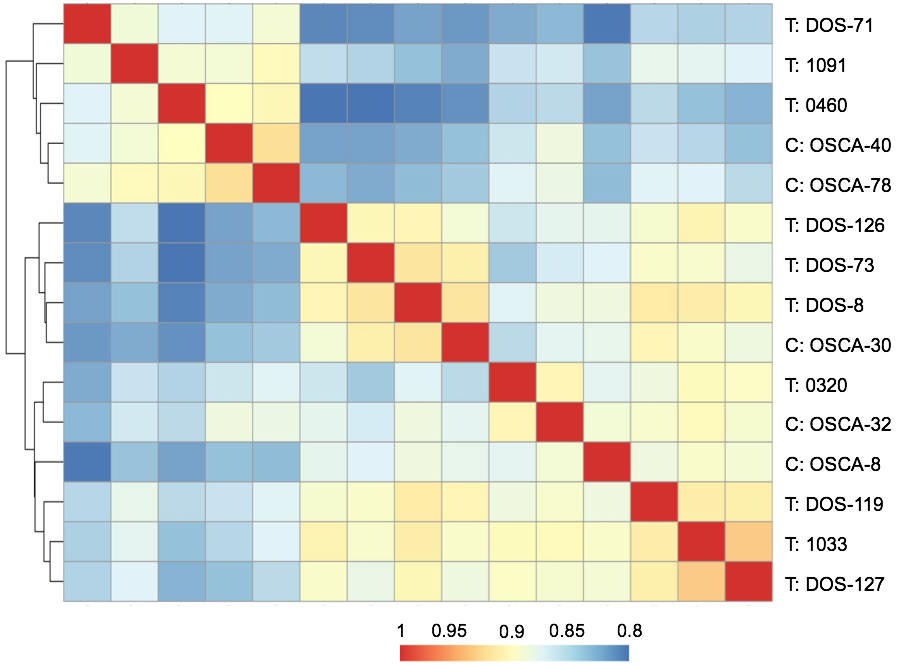


**Fig. S2**

**Unsupervised hierarchical clustering of mRNA-Seq data from canine osteosarcoma samples aligned to the genome of the dingo *Canis lupus dingo*.**

Samples: tissues (T) or cell lines (C) of set 1.
