# Supplementary material for: S100A4 mRNA-protein relationship uncovered by measurement noise reduction
Source: J Mol Med (Berl). 2020 Apr 15;98(5):735–49. doi: 10.1007/s00109-020-01898-8 (PMC7241963; doi:10.1007/s00109-020-01898-8)
Supplement: Supplementary file 11 — (DOCX 139 kb) [file 109_2020_1898_MOESM11_ESM.docx]

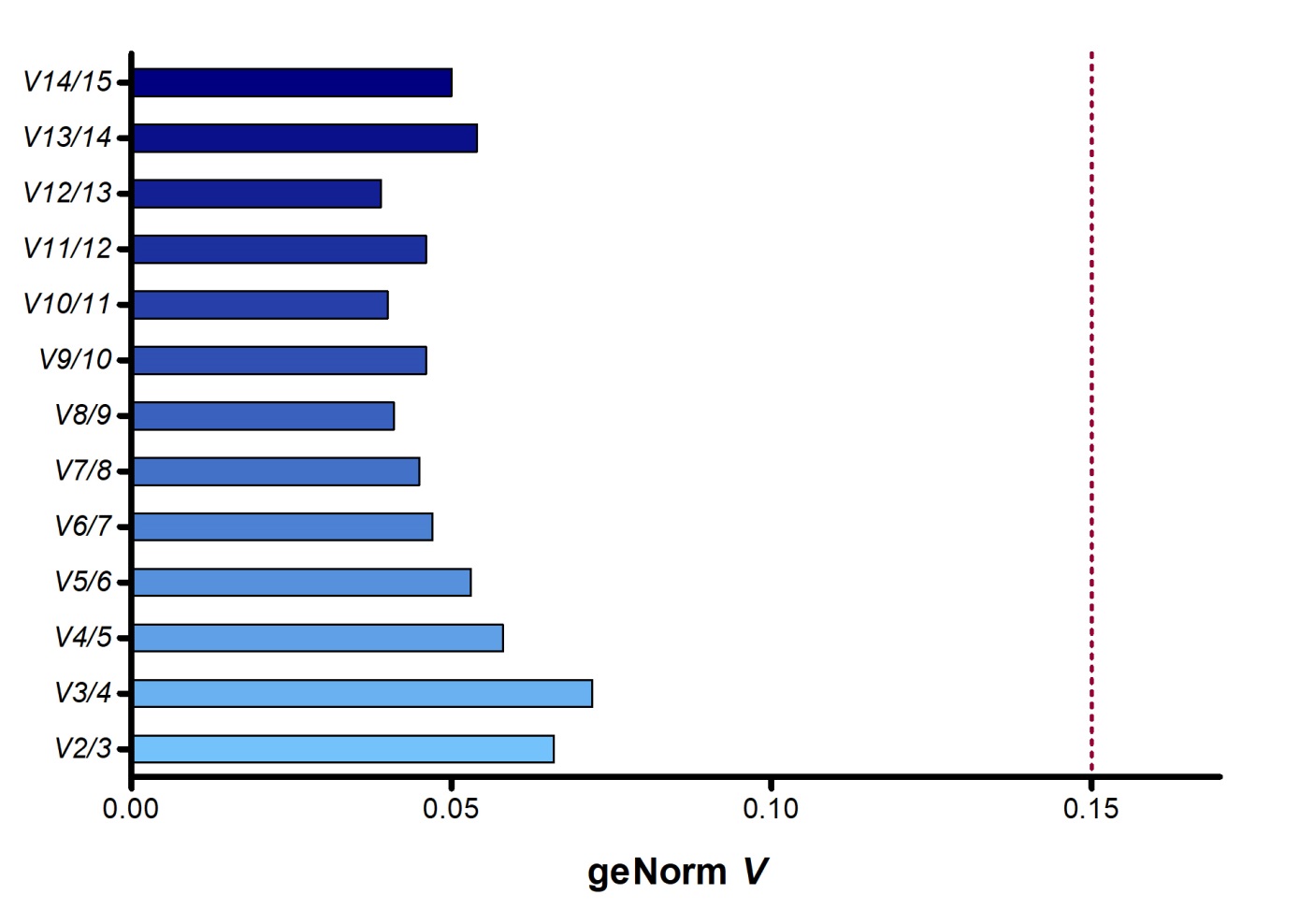


**Fig. S3**

**Two sequences are sufficient for normalisation of RT-qPCR data in the context of canine osteosarcoma.** The optimal number of normalisers was evaluated by the geNorm software [1]. The tool calculated *V* values as a pairwise variation, *V_n_/V_n+1_*, between two consecutively ranked normalisation factors (*NF*) after the stepwise addition of the next stable sequence of the list (*NF_n_* and *NF_n+1_*), where *n* indicates the number of the most stable reference sequences. The dashed line indicates the recommended cut-off for pairwise variation of 0.15 below which the inclusion of a further sequence would not add to the stability of the normalisation factor.

**References**

[1] Vandesompele J, De Preter K, Pattyn F, Poppe B, Van Roy N, De Paepe A, et al. Accurate normalization of real-time quantitative RT-PCR data by geometric averaging of multiple internal control genes. Genome Biol. 2002;3:RESEARCH0034.
