# Supplementary material for: S100A4 mRNA-protein relationship uncovered by measurement noise reduction
Source: J Mol Med (Berl). 2020 Apr 15;98(5):735–49. doi: 10.1007/s00109-020-01898-8 (PMC7241963; doi:10.1007/s00109-020-01898-8)
Supplement: Supplementary file 12 — (DOCX 702 kb) [file 109_2020_1898_MOESM12_ESM.docx]

**
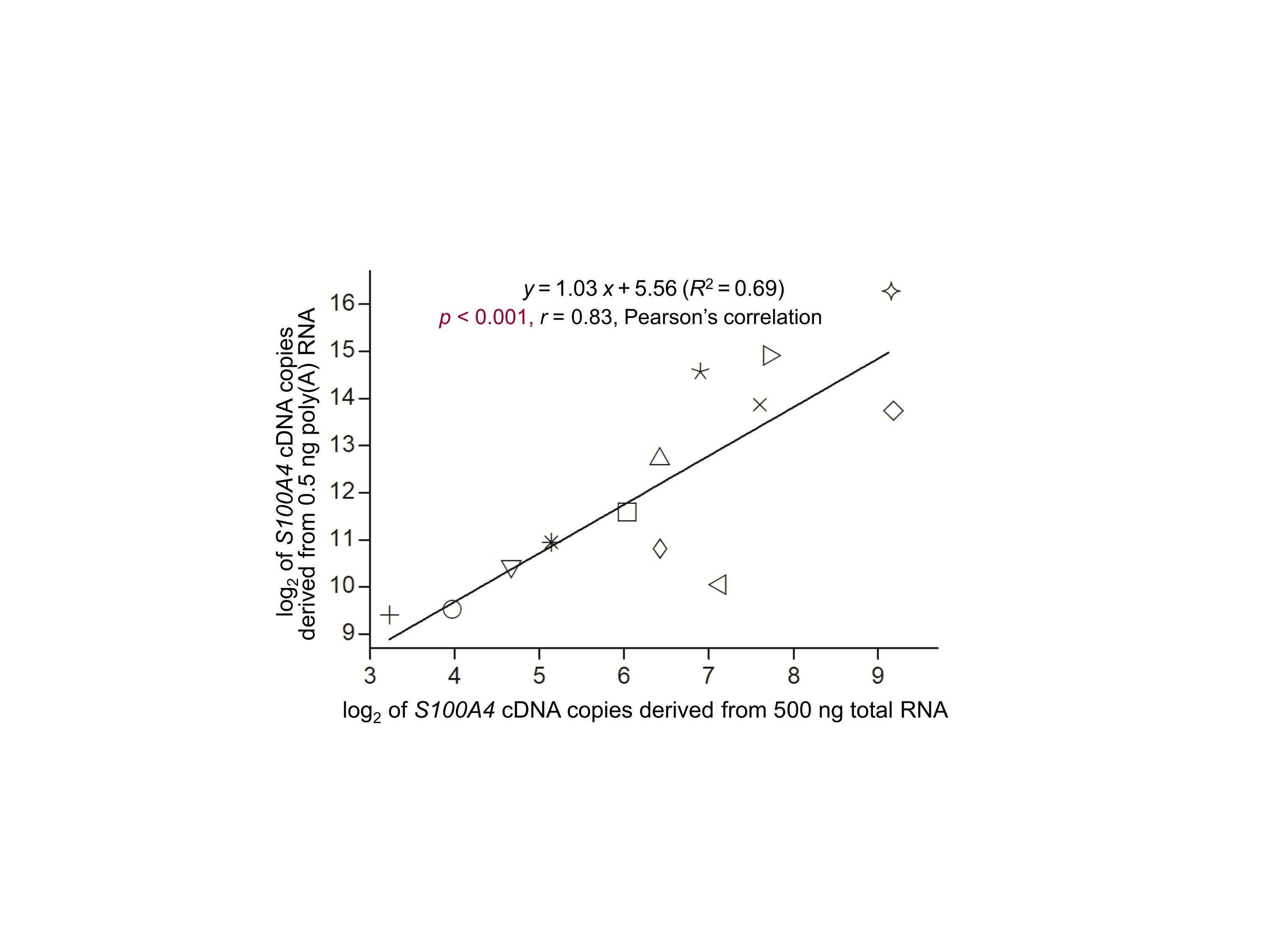
**

**a**

**b**


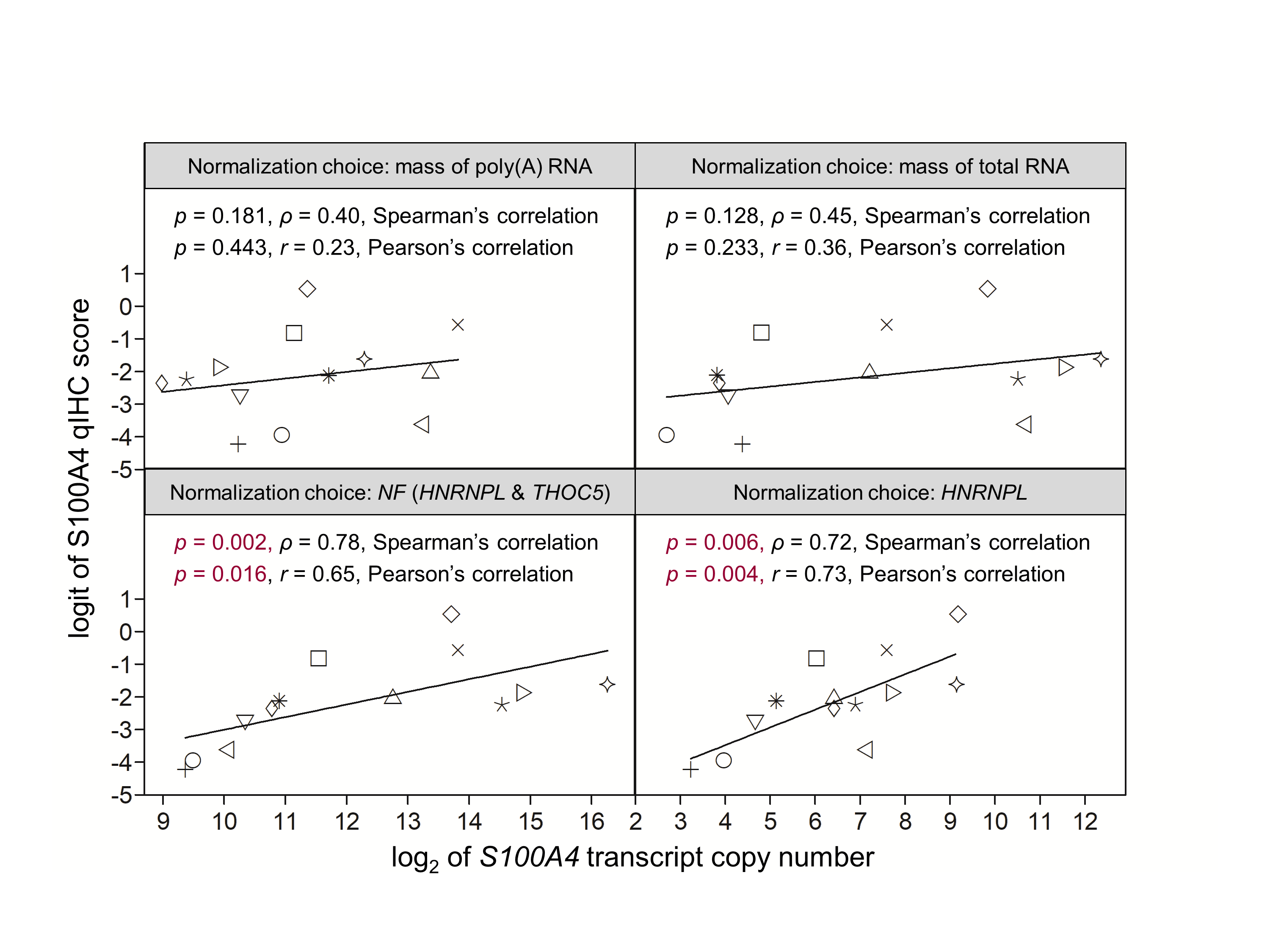


**Fig. S4**

**Association between transcript and protein expression levels of canine *S100A4* can be uncovered by normalisation with consecutive exons optimised for the biological context.**

**a** Both total cellular RNA or its poly(A) RNA fraction are suitable template types for measuring overall transcript expression of canine *S100A4*. For sample identities see Fig. 5d.

**b** We failed to detect a significant relationship between the two expression levels of *S100A4* when normalising with RNA mass used as template in cDNA synthesis (upper left: 500 pg poly(A) RNA or upper right: 500 ng total RNA), in contrast to normalisation with the geometric mean of the consecutive-exon pairs of *HNRNPL* and *THOC5* at mRNA-template level (lower left) or just *HNRNPL* in case of total RNA templates (lower right).

Protein abundance was expressed as the proportion of tumour area stained (qIHC score). Out of the two replicate qIHC scores measured for sample #0649, only the value that better matched the overall mRNA to protein correlation was presented. Samples: validation cohort (set 2, Table S2).

*p:* corresponding significance value (in red: *p* ≤ 0.05);

*ρ*: Spearman's rank correlation coefficient;

*r*: Pearson’s correlation coefficient
