# Supplementary material for: S100A4 mRNA-protein relationship uncovered by measurement noise reduction
Source: J Mol Med (Berl). 2020 Apr 15;98(5):735–49. doi: 10.1007/s00109-020-01898-8 (PMC7241963; doi:10.1007/s00109-020-01898-8)
Supplement: Supplementary file 13 — (DOCX 429 kb) [file 109_2020_1898_MOESM13_ESM.docx]

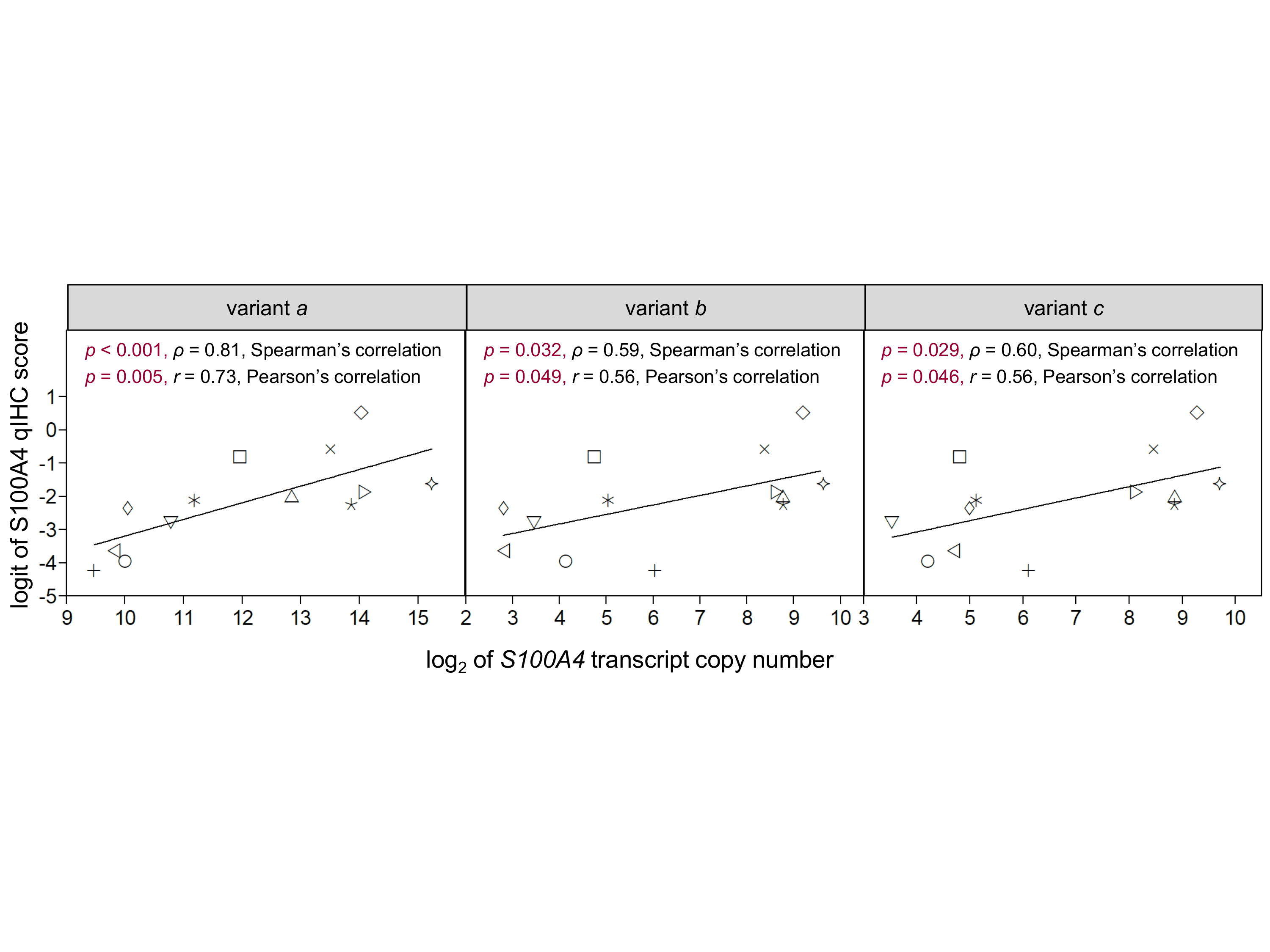
**Fig. S5**

**The major transcript variant *a* of *S100A4* better correlates with the qIHC score of the S100A4 protein compared to its mRNA species *b* & *c*.** Expression of the three mRNA species was normalised by the normalisation factor derived by calculating the geometric average of consecutive-exon pairs of the gene combination *HNRNPL* and *THOC5*. Protein abundance was expressed as qIHC score representing the proportion of tumour area stained. Samples: set 2 (Table S2).  Out of the two replicate qIHC scores measured for sample #0649, we included only the value that better fitted to the regression line in Fig. 7.

*p:* corresponding significance value; *ρ*: Spearman's rank correlation coefficient.
