# Supplementary material for: S100A4 mRNA-protein relationship uncovered by measurement noise reduction
Source: J Mol Med (Berl). 2020 Apr 15;98(5):735–49. doi: 10.1007/s00109-020-01898-8 (PMC7241963; doi:10.1007/s00109-020-01898-8)
Supplement: Supplementary file 14 — (DOCX 83 kb) [file 109_2020_1898_MOESM14_ESM.docx]

**Table S1. Tissue samples and cell lines of canine osteosarcoma used for mRNA-Seq analysis (material set 1)**

| Sample | | Breed (gender) | Age at diagnosis (years) | Origin | Histo-pathologial subtype | Treatment | Survival since diagnosis (months) | Proli-feration type^‡^ | RNA isolation protocol^§^ |
| --- | --- | --- | --- | --- | --- | --- | --- | --- | --- |
| Osteosarcoma tissues |  |  |  |  |  |  |  |  |  |
| 0320 | | mixed (♀) | 12.5 | distal femur/ proximal tibia | osteochondro-sarcoma | no^†^ | 0.00 | unknown | 1 |
| 0460 | | Landseer (♀) | 6.2 | distal tibia | osteoblastic | carboplatin | 1.63 | unknown | 1 |
| 1033 | | Boxer (♀) | 7.1 | distal tibia/lung metastasis | teleangiectatic (lung metastasis) | carboplatin | unknown | unknown | 1 |
| 1091 | | Leonberger (♀) | 7.0 | distal tibia | osteoblastic | amputation | unknown | unknown | 1 |
| DOS-8 | | Rottweiler (♂) | 2* | left proximal humerus | osteoblastic variant | unknown | unknown | aggressive | 2 |
| DOS-71 | | Golden Retriever (♀) | 6.7 | unknown | osteoblastic | SOC | 16.00 | aggressive | 2 |
| DOS-73 | | Golden Retriever (♀) | 2.5 | right proximal tibia | osteoblastic | palliative | 1.48 | less aggressive | 2 |
| DOS-119 | | Great Pyrenees (♀) | 8.4 | right distal radius | osteoblastic | no^†^ | 0.00 | unknown | 2 |
| DOS-126 | | Great Dane (♂) | 4.6 | left distal radius | not analysed | SOC | 2.47 | unknown | 2 |
| DOS-127 | | English Mastiff (♀) | 7.2 | front leg | osteoblastic | palliative | 0.00 | unknown | 2 |
| Primary cultures of osteosarcoma cells |  |  |  |  |  |  |  |  |  |
| OSCA-8 | | Rottweiler (♂) | 2* | left proximal humerus | osteoblastic variant | unknown | unknown | aggressive | 3 |
| OSCA-30 | | German Shepherd (♂) | 8.7 | left distal radius | osteoblastic | SOC | 20.61 | less aggressive | 3 |
| OSCA-32 | | Great Pyrenees (♀) | 8.7 | left distal radius | fibroblastic OS variant | SOC | 11.01 | less aggressive | 3 |
| OSCA-40 | | Saint Bernard (♀) | 5.8 | right distal femur | osteoblastic | amputation | 1.18 | aggressive | 3 |
| OSCA-78 | | German Shepherd (♂) | 9.5 | right distal femur | fibroblastic/ osteoblastic mix | SOC | 2.50 | aggressive | 3 |

*Might be younger (~1 y); ^†^Euthanized at diagnosis; ^‡^Information on the level of tumour aggressiveness was taken from: Scott, M. C*. et al*. *Bone* **49**: 356-67 (2011); ^§^1: RNeasy Fibrous Tissue Mini Kit (Qiagen, Hilden, Germany), 2: TRIzol™ Reagent (Thermo Fisher Scientific, Waltham, MA, USA), 3: RNeasy Mini Kit (Qiagen); SOC: standard-of-care chemotherapy
