# Supplementary material for: S100A4 mRNA-protein relationship uncovered by measurement noise reduction
Source: J Mol Med (Berl). 2020 Apr 15;98(5):735–49. doi: 10.1007/s00109-020-01898-8 (PMC7241963; doi:10.1007/s00109-020-01898-8)
Supplement: Supplementary file 15 — (DOCX 114 kb) [file 109_2020_1898_MOESM15_ESM.docx]

**Online Supplemental Methods**

**Materials and methods**

**Biological material**

Normal canine osteoblasts (Cell Applications Inc., San Diego, CA, USA; catalogue no. Cn406-05) were cultured in a canine osteoblast medium (Cell Applications Inc., catalogue no. Cn417-500).

Samples of spontaneously developed osteosarcoma were collected from large- and medium-sized dog breeds during routine medical treatment at the College of Veterinary Medicine of the University of Minnesota or at the Animal Hospital of the University of Veterinary Medicine of Vienna, following the rules of the local ethical committees. For transcript profiling by mRNA-Seq or RT-qPCR, experimental specimens were grouped into two sets, termed set 1 and set 2, respectively. Set 1 included ten osteosarcoma tissues (#0320, #0460, #1033, #1091, DOS-8, -71, -73, -119, -126 and -127) and five primary cell lines (OSCA-8, -30, -32, -40 and -78) derived from newly diagnosed patients prior to treatment with cytotoxic chemotherapy drugs (Table S1). The processing of tumour specimens and generation of primary cell cultures has been described earlier [1, 2]. Set 2 comprised exclusively tumour tissue (*n* = 13, Table S2). Clinical and pathological data of the sample cohort are listed in Tables S1 and S2, respectively. Aliquots of osteosarcoma tissue were snap-frozen at -170 °C or preserved in an RNA-stabilising buffer (RNAlater; Qiagen, Hilden, Germany) and stored in the gas phase over liquid nitrogen.

**Short Tandem Repeat DNA Profile Analysis for cell line authentication**

To exclude cross-contamination and other causes of misidentification, the five osteosarcoma cell lines used in this study were genotyped at 15 short tandem repeat (microsatellite) loci. Profiling of microsatellites was performed by IDEXX BioResearch ([www.idexxbioresearch.com](http://www.idexxbioresearch.com)). For authenticating genetic relatedness between donor (tumour tissue or original cell line) and the cell line at the respective passage used for this study, we adopted a threshold of at least 80 % matching alleles [3]. This percentage match limit was based on the idea that the microsatellite profile from a malignant tissue can vary with loss of heterozygosity and an elevated incidence of microsatellite instability and complexity and allowed for some genetic drift with increasing passage number of a cell line. The match value was obtained by dividing the number of shared alleles by the total number of alleles in the questioned profile.

Our authenticating genotype data included one to two deviating microsatellite alleles, mostly loss of heterozygosity rather than a change in the allele length (Data S1).

**Extraction of total RNA**

RNA of normal osteoblasts and a part of the tissues of sample set 1 was isolated using the TRIzol™ Reagent, a monophasic solution of phenol and guanidine isothiocyanate (Thermo Fisher Scientific, Waltham, MA, USA).  RNA of tissues of set 2 and cultured osteosarcoma cells of set 1 was extracted with a silica-based membrane combined with micro-spin technology.

For TRIzol™ extraction from osteoblast cells [3] or osteosarcoma tissue (50 to 100 mg), one millilitre of the reagent was used. Subsequently, samples were supplemented with 0.2 ml chloroform, vortexed vigorously for 15 s and incubated at room temperature for 10 min. Following centrifugation at 12.000 × *g* at 4 °C for 15 min, the RNA-containing upper phase was transferred into a fresh 1.5 ml tube and precipitated with 0.5 ml isopropyl alcohol. Once again, the samples were incubated at room temperature for 10 min, followed by centrifugation at 12.000 × *g* at 4 °C for 10 min. The RNA pellet was washed once with 1 ml 70 % ethanol and centrifuged at 7.500 × *g* at 4 °C for 5 min. Finally, the RNA pellet was air dried for 5 min and dissolved in diethylpyrocarbonate (DEPC)-treated water.

For spin column-based extraction of tissue RNA, the RNeasy Fibrous Tissue Mini Kit (Qiagen) was used. Up to 30 mg of osteosarcoma tissue was transferred to bead-beating tubes filled with 1 g ceramic beads (1.4 mm diameter; Peqlab Biotechnologie GmbH, Erlangen, Germany) and 300 μl Buffer RLT (guanidine thiocyanate-containing lysis buffer) supplemented with β-mercaptoethanol (10 µl per 1 ml buffer). The tissue was disrupted in the MagNA Lyser Instrument (Roche Diagnostics, Rotkreuz, Switzerland) using three rounds of centrifugation at 6,500 rpm for 20 s interrupted by cooling for 2 min. Further steps including removal of contaminating genomic DNA by on-column DNase I digestion were performed according to the protocol of the kit manufacturer.

RNA from 10^6^ to 10^7^ cultured osteosarcoma cells was extracted using the RNeasy Mini Kit (Qiagen) according to the kit protocol.

The concentration of RNA subjected to mRNA-Seq profiling was measured using the fluorescent dye-based Quant-iT^™^ RiboGreen^®^ RNA Assay Kit (Thermo Fisher Scientific). Its integrity was assessed by capillary electrophoresis on the 2100 Bioanalyzer Instrument (Agilent Technologies, Santa Clara, CA, USA). Next-generation sequencing libraries were prepared only from samples that exhibited an RNA Integrity Number (RIN) value of at least 8 and a quantity higher than 1 μg.

Concentration and purity of RNAs subjected to profiling by RT-qPCR were determined on the NanoDrop™ 2000c spectrophotometer (Thermo Fisher Scientific). Minimum RNA intactness assessed at the 4200 TapeStation System (Agilent Technologies) was set at a value of ≥ 6.4 RNA integrity number equivalent (RIN^e^).

**Library preparation and sequencing**

Sequencing libraries focused on poly(A) RNA were generated from 1 µg total RNA using the TruSeq RNA library preparation kit v2 (Illumina, San Diego, CA, USA). The size distribution of the final library was validated using capillary electrophoresis and quantified by the Quant-iT™ PicoGreen™ dsDNA Assay Kit (Thermo Fisher Scientific) and via qPCR. The indexed libraries were normalised, pooled and size selected to 320 bp ± 5 % using the LabChip XT for nucleic acid fractionation (Caliper Life Sciences, Hopkinton, MA, USA). TruSeq libraries were hybridised to a paired end flow cell and individual fragments were clonally amplified by bridge amplification on the Illumina cBot system. Once the clustering was complete, the flow cell was loaded and sequenced with sequencing-by-synthesis technology on the HiSeq^TM^ 2000 Sequencing System (Illumina).

**Bioinformatics pipeline for mRNA-Seq data**

Primary analysis and de-multiplexing of mRNA-Seq data was performed using the CASAVA software 1.8.2 (Illumina).

For hierarchical clustering of expression patterns, sequence reads were aligned to the reference genome of the dog, *Canis lupus familiaris,* or the dingo, *Canis lupus dingo* (Ensembl genome browser assemblies CanFam3.1 or ASM325472v1, respectively) using Spliced Transcripts Alignment to a Reference (STAR, version 2.7.3; [4]; <https://github.com/alexdobin/STAR/releases>).

For selection of stable exons, we used TopHat (<https://ccb.jhu.edu/software/tophat/index.shtml)>, another popular, but less recent splice-aware aligner.

The FASTQ files were mapped with Tophat [5] and compared to the reference annotation for the dog (CanFam3.73) with help of the Cufflinks packages using default parameters [6]. After mapping, reads with multiple matches to the genome were removed to only keep uniquely mapped reads using samtools [7]. Genes and exons were annotated based on the reference annotation of the Ensembl Genome Browser (genomic sequence version 3.1, release 73; [8]). DEXSeq [9] python scripts were used to produce a ﬂattened version of this ﬁle, to collapse exons across different isoforms and to extract the uniquely mapped reads per exon. Gene expressions represented by all mapped reads were clustered by the R package pheatmap [10] using Spearman's *ρ*.

**Expression stability ranking of single and neighbour exons based on *CV***

The stability of exonic sequences in the mRNA-Seq expression data was ranked in ascending order based on the coefficient of variation (*CV*) defined as the ratio of the standard deviation to the mean [11]. Individual *CV* values were calculated for single exons and three exon-neighbour combinations, namely a pair of direct neighbours (*i* and *i* + 1), a trio of direct neighbours (*i*, *i* + 1 and *i* + 2) and an exon paired with its neighbour after next (*i* and *i* + 2). Calculation was performed using R package version 3.5.5 ([www.R-project.org/](https://www.r-project.org/)).

**Enrichment analysis for gene ontology (GO) terms**

The most stable genes were annotated by one or more GO terms using the Blast2Go tool ([12]; [www.blast2go.com](http://www.blast2go.com)). Briefly, Standard Protein BLAST (<https://blast.ncbi.nlm.nih.gov/Blast.cgi?PAGE=Proteins>) was performed against the non-redundant database of NCBI for all mammals (taxon: 40674). Only the first 20 alignments passing the *E* value cutoff of 1.0E-3 with a coverage of at least 90 % against the subject sequences were considered for annotation. The GO terms retrieved by InterPro scanning at the web server of the European Bioinformatics Institute () were converted and merged with the annotation. The annex function was employed to assign the GO terms obtained by GO mapping to the query sequences. Finally, “slimming” was carried out to identify the most representative biological processes that were subsequently subjected to enrichment analysis. The annotations of all known canine proteins were used as a background list to create a 2 × 2 contingency table for calculating the Fisher’s Exact Test [13] in Microsoft Excel (<https://udel.edu/~mcdonald/statfishers.xls>). Enriched GO terms with a false discovery rate of less than 0.01 were reported. Concordance of the enrichment results was evaluated at the GeneMania database ([14, 15]; <http://genemania.org>) using human genomics and proteomics data as orthologous substitute for the dog and the "GO biological process" as the weighting method.www.ebi.ac.uk/interpro/) were converted and merged with the annotation. The annex function was employed to assign the GO terms obtained by GO mapping to the query sequences. Finally, “slimming” was carried out to identify the most representative biological processes that were subsequently subjected to enrichment analysis. The annotations of all known canine proteins were used as a background list to create a 2 × 2 contingency table for calculating the Fisher’s Exact Test [13] in Microsoft Excel (<https://udel.edu/~mcdonald/statfishers.xls>). Enriched GO terms with a false discovery rate of less than 0.01 were reported. Concordance of the enrichment results was evaluated at the GeneMania database ([14, 15]; <http://genemania.org>) using human genomics and proteomics data as orthologous substitute for the dog and the "GO biological process" as the weighting method.

**Oligo dT-based mRNA isolation for RT-qPCR**

The poly(A) RNA fraction was extracted from total cellular RNA using the NEBNext^®^ Poly(A) mRNA Magnetic Isolation Module (New England Biolabs, Ipswich, MA, USA). The kit facilitates anchoring of mRNAs to 1 μm paramagnetic oligo (dT)_25_ beads. Quantity of poly(A) RNA was determined on the DS-11 FX+ instrument (DeNovix, Wilmington, DE, USA) following the optimised Qubit™ RNA HS Assay [16]. A mean concentration was obtained by three measurements of a sample’s aliquot.

**cDNA synthesis**

First-strand cDNA synthesis was performed in a 20-μl volume using the Transcriptor High Fidelity cDNA Synthesis Kit (Roche Life Science, Vienna, Austria). The reaction contained 1 × Transcriptor RT reaction buffer, 1 mM dNTP mix, 60 µM random hexamers or 2.5 μΜ oligo(dT)_18_ primer in case of the poly(A)-RNA template, 20 U Protector RNase inhibitor, 10 U Transcriptor Reverse Transcriptase, and 500 ng total RNA or 500 pg poly(A) RNA. To meet the pronounced intrinsic variability of RT [17, 18], two parallel reactions were produced. Putative contamination with genomic DNA was monitored by a minus-reverse transcriptase control produced by replacing the enzyme with nuclease-free water. RT was performed at 55 °C for 60 min and terminated at 85 °C for 5 min using the MJ Research PTC-200 Thermal Cycler (Bio-Rad, Hercules, California, USA). The undiluted cDNAs were stored at -20 °C until further analysis.

**Detection of qPCR assay inhibitors (SPUD assay)**

To detect qPCR assay inhibition, a spiked synthetic sequence that lacks homology with mammalian DNA was amplified (SPUD assay [19]). The 15 -µl reaction contained 10 × buffer B2 (Solis Biodyne, Tartu, Estonia), 3.5 mM MgCl_2_, 200 nM of each dNTP, 250 nM of each primer, 250 nM hydrolysis probe, 10^5^ copies of the spike, 1 unit HOT START DNA polymerase (HOT FIREPol^®^ DNA Polymerase, Solis Biodyne) and 6 µl of 1:6 diluted cDNA. Nuclease-free water was used to determine the "unaffected” *Cq* value. The cycling conditions on the Rotor-Gene 6000 qPCR system (Corbett Life Science, Concorde, Australia) comprised 15 min at 95 °C for polymerase activation and 40 amplification cycles of 15 s at 95 °C and of 40 s at 58 °C. A difference between *Cq*s of cDNA and control of < 1 was considered as evidence for lack of inhibition.

**qPCR**

# Oligonucleotide sequences of the qPCR assays (Table S3) were designed using the program Primer Express 2.0 (Applied Biosystems, Foster City, CA, USA). The chance of amplifying co-isolated genomic DNA was reduced by designing a PCR product that spanned an exon-intron boundary or flanked an intron of at least 750 bp. Putative dimerisation of primers was evaluated with the primer analysis software NetPrimer (Premier Biosoft International, Palo Alto, CA, USA; [www.premierbiosoft.com/netprimer/](http://www.premierbiosoft.com/netprimer/)). The secondary structure of the PCR amplicon was predicted on the Mfold Web Server ([20]; <http://unafold.rna.albany.edu/?q=mfold/DNA-Folding-Form>). Amplicon specificity was evaluated by the NCBI tool Primer-BLAST [21] using the "non-redundant" database of the dog (taxid number: 9615).

The qPCR was performed in a 15-μl volume composed of 1 × PCR buffer B2 (Tris-HCl, (NH_4_)_2_SO_4_ and Tween-20; Solis Biodyne), 1 × dNTP mix that partially replaced dTTP with dUTP to combine strong amplification with the option of PCR product carryover prevention by using uracil-DNA N-glycosylase (0.2 mM of each dATP, dCTP and dGTP, 0.08 mM dUTP and 0.12 mM dTTP; Solis Biodyne), 0.4 × EvaGreen I dye (Biotium, Fremont, CA, USA) or 200 nM hydrolysis probe (Integrated DNA Technologies, Leuven, Belgium) depending on the assay, 3.5 mM MgCl_2_, 200 nM of each primer, 1 U HOT FIREPol^®^ DNA Polymerase (Solis Biodyne) and 1.5 µl or 6 µl of diluted cDNA. For target quantification in total cellular RNA or poly(A)-RNA templates, cDNAs were ten- or six-fold diluted, respectively. All qPCR assays were run in duplicates (or triplicates in case of the *S100A4* transcript variants), included a minus-RT control and a no-template control to rule out cross contamination of reagents and surfaces. Amplification and monitoring of fluorescence were performed on the Corbett Rotor-Gene 6000 Real Time PCR System (Qiagen) operated by the software version Rotor-Gene Q 2.1.0.9. Cycling conditions consisted of an initial 15-min incubation step at 95 °C for polymerase activation and template denaturation, followed by 50 cycles of 95 °C denaturation for 15 s, 60 °C annealing for 20 s and 72 °C elongation for 20 s. Finally, a dissociation curve was recorded over the range of 60 to 95 °C at increments of 1 °C every 5 s. In case of the probe-based qPCR format, amplification was achieved over 50 cycles consisting of a 15 s denaturation step at 95 °C and combined annealing and elongation for 60 s at 60 °C. Increasing the cycle number of dye-based qPCR from 35-40 to 50 helps to achieve similar fluorescence endpoints [22], thus positively affects the melting profile of the amplicon. A qPCR assay was considered unaffected by genomic co-amplification if the minus-RT control produced an efficiency-adjusted Δ*Cq* value of at least 5.

# In addition to the amplicon melting profile, specificity of qPCR primers was validated by electrophoresis on a 1 % agarose gel (Fig. S1) using 1 × sodium borate buffer as a simple buffer for rapid and cost-effective DNA electrophoresis [23]. The gel was stained with GelGreen™ Nucleic Acid Stain (Biotium), a sensitive, stable and environmentally safe green fluorescent nucleic acid dye (8.75 μl dye per 100 ml gel). DNA fragment size was determined with the marker "100 bp DNA Ladder" (Solis Biodyne).

RT-qPCR assay design complies with the essential requirements of the Minimum Information for Publication of Quantitative Real-Time PCR Experiments (MIQE) guidelines [24].

**Sanger sequence analysis**

Sanger capillary sequencing of PCR products was performed with forward and reverse amplification primers (Table S4). In brief, amplicons were separated by agarose gel electrophoresis, purified with the Hi Yield^®^ Gel/PCR DNA Fragment Extraction Kit (SLG, Gauting, Germany) and sequenced at LGC Genomics GmbH (Berlin, Germany). Chromatograms were edited in the DNA sequencing software CodonCode Aligner (version 3.7.1.2; CodonCode Corporation, Centerville, MA, USA).

**Determination of qPCR amplification efficiency and outlier treatment**

In case of target sequences of at least moderate abundance, efficiency of qPCR amplification (*E*) can be determined from raw fluorescence data without the need of a standard curve. For each well, an individual efficiency was calculated from the exponential phase of the raw (*i.e.* not baseline-corrected) amplification curve using the Real-time PCR Miner ([25]; <http://ewindup.info/miner/)>. The mean of individual efficiencies, *E_fi_,* served to "compensate” *Cq* values measured at an amplification efficiency of less than 100 % according to the term *Cq* × log_10_ (*E_fi_* + 1) /log_10_(2) [26]. Following efficiency-correction of *Cq* values, outliers from quadruplicate RT-qPCR measurements, *i.e.* qPCR replicates for both cDNA duplicates, were identified and handled as follows. In case of quadruplicate *Cq* values, we removed the technical replicate that caused a standard deviation of more than 0.5 cycles. If the means of the qPCR duplicates run for each of the two cDNA replicates differed by more than one cycle, the cDNA replicate that exhibited the highest deviation from the global average of the sample cohort was removed from analysis (*n* = 7: qPCR-based validation of reference exons).

**RT-qPCR measurement of exon’s expression stability**

The two steps of an RT-qPCR assay were run in duplicates starting with the RT reaction. The average of the duplicate’s raw *Cq* values was efficiency-adjusted and assessed for expression stability using the four common statistical algorithms, geNorm [27] implemented in the qbase+ software 3.1 (Biogazelle, Ghent, Belgium; [28]; [www.qbaseplus.com](http://www.qbaseplus.com)), the Microsoft Excel-based programs NormFinder ([29]; <https://moma.dk/normfinder-software>) and BestKeeper ([30]; [www.gene-quantification.de/bestkeeper.html](http://www.gene-quantification.de/bestkeeper.html)) as well as the Comparative Δ*Cq* method [31] accessed via the web-based tool RefFinder ([32]; <http://www.heartcure.com.au/reffinder/>). The stability scores of the algorithms were compiled into a final rank using the RankAggreg package in R ([33]; <https://CRAN.R-project.org/package=RankAggreg>), version 3.4.4 for Windows, using the cross-entropy Monte Carlo algorithm with Spearman’s footrule distance. The R code for rank aggregation analysis is provided as File S1.

The minimum gene set for RT-qPCR normalisation was determined by the geNorm algorithm [27]. The software sorts genes in ascending order according to their expression stability measure (*M*), computes a *NF* for each gene set using the geometric mean of their expression values and determines the optimal number of normalisers based on pairwise variation (*V_n_/V_n+1_*) between two sequential factors, *NF_n_* and *NF_n+1_*. The common cut-off value of *V_n_/V_n+1_* < 0.15 determined the lowest number of genes for accurate normalisation [27]. This minimum gene number was adopted to compose the *NFs* for the other stability algorithms. In another round of stability assessment with the RankAggreg package we computed consensus ranks for the single genes and the added four *NF*s.

Co-regulated gene relationship of the gene pair composing the best *NF* was evaluated based on information for the dog contained in version 7.3 of the gene co-expression database COXPRESdb ([34]; http://coxpresdb.jp).

**Assignation of biochemical processes using gene ontology classification**

Molecular functions of the gene pair regarded as the most appropriate *NF* were derived from the Gene Ontology Database (release: 1^st^ of January 2019; [35]; <http://amigo.geneontology.org/amigo>).

***In silico* analysis of alternative polyadenylation in the 3' UTR of canine *S100A4* mRNA**

Hexanucleotide motifs signalling canonical (AAUAAA) as well non-canonical (AUUAAA, AGUAAA, or UAUAAA) polyadenylation [36] were used as query sequences for *in silico* analysis by "Poly(A) Signal Miner" integrated in DNA Functional Site Miner ([37]; <http://dnafsminer.bic.nus.edu.sg>).

**Rapid Amplification of 3’ cDNA Ends (3' RACE)**

The 3’ untranslated region of canine *S100A4* was amplified by 3′ RACE-PCR [38]. A tagged and anchored oligo(dT) primer, shortly termed Oligo(dT)-Anchor Primer (Table S4), primed the conversion of poly(A) RNA into first-strand cDNA and introduced partially overlapping target sites for two artificial nested PCR primers. The primer’s anchor represented a mixture of oligonucleotides carrying a non-T nucleotide (*i*.*e*., A, C, or G) at the 3’ end following the dT stretch. This forces the primer to bind to the 5’ start site of the poly(A) tail, thus excluding the influence of poly(A)-tail length.

For cDNA synthesis, 500 pg poly(A) RNA template was incubated together with 2.5 µM Oligo dT-Anchor Primer at 65 °C for 10 min to denature secondary structure, rapidly chilled on ice for at least 1 min, mixed with the remaining components supplied by the Transcriptor High Fidelity cDNA Synthesis Kit (Roche Life Science) and reverse transcribed at 47 °C for 1 h. In the nested PCR step, the binding sites introduced by the 5’ artificial tail sequence allowed targeting by partially overlapping nested PCR primers termed Outer-R and Inner-R. They were used in combination with a pair of nested forward primers amplifying all three *S100A4* transcript variants or with nested forward primers that specifically amplified transcript *c*. Nested PCR was performed in a 20-μl reaction consisting of 1 × PCR buffer B2 (Tris-HCl, (NH_4_)_2_SO_4_ and Tween-20; Solis Biodyne), 1 × dNTP mix (see 2.10), 1.5 mM MgCl_2_, 5 nM of outer primer, 250 nM of inner primer, 1 U HOT FIREPol^®^ DNA Polymerase (Solis Biodyne) and 1.5 µl cDNA. Amplification was performed over 20 cycles at the annealing temperature of 65 °C followed by 35 cycles at 55 °C.

**Counting *S100A4* mRNA copies by digital PCR (dPCR)**

The copy number concentration of a cDNA target sequence was determined by dPCR [39] on the Applied Biosystems QuantStudio™ 3D Digital PCR System (Thermo Fisher Scientific). Twelve microliters of master mix consisting of 1 × QuantStudio™ 3D Digital PCR MasterMix, 200 nM of each primer and 200 nM hydrolysis probe (Table S3) was mixed with 6 µl of a cDNA dilution adjusted according to qPCR measurement. A 14.5-µl aliquot of the mixture was loaded to the dPCR chip. Thermal cycling on the GeneAmp^®^ PCR System 9700 thermal cycler (Applied Biosystems) was performed according to instruction of the manufacturer (initial denaturation: 96 °C for 10 min, 45 amplification cycles: denaturation at 98 °C for 30 s and annealing/elongation at 60 °C for 2 min, final incubation: 60 °C for 2 min). The endpoint fluorescence in the chip's partitions read using the QuantStudio 3D Chip Reader (software version 3.0; Thermo Fisher Scientific) delivered the number of target-positive wells, the number of wells without DNA (negative for the reporter fluorophore FAM™, but positive for the passive reference dye ROX™) and empty (ROX-negative) wells. Data considered marginal or failing in initial quality assessment were flagged for secondary review using the QuantStudio™ 3D AnalysisSuite™ software (application version: 3.1.2-PRC-build-03, algorithm version: 4.4.10). In this step, positive signals were limited by manually setting the fluorescence threshold next to the bottom of the main peak depicted in the menu “well counts per fluorescence”. The Poisson-Plus Model [40] was used to determine the number of target copies per microliter from positive and negative partitions. Finally, the *Cq* values of experimental samples obtained with the qPCR assays for the *S100A4* transcript variants and their consensus were transformed into transcript copy numbers based on dPCR counts of the sample that exhibited the highest expression of the major *S100A4* transcript variant (sample #2097).

**Quantitative immunohistochemistry (qIHC) for S100A4**

The area fraction of (brown) colour pixels resulting from immunohistochemical staining against S100A4 was determined by qIHC. To reduce intra- and inter-observer variability in the assessment, the tissue distribution of the target protein was automatically scored by quantitative image analysis [41, 42]. In detail, paraffin sections were rehydrated and blocked by 1.5 % normal goat serum to minimise unspecific binding of the primary antibody. Sections were heated in 0.1 M citrate buffer (pH 6) for 30 min for heat-induced epitope retrieval and incubated over night with the monoclonal anti-S100A4 antibody produced in the mouse (Sigma-Aldrich, Vienna, Austria; catalogue number AMAb90599, Prestige Antibodies^®^, clone CL0240, 1:3,000 dilution). Antibodies of this series were developed and validated by the Human Protein Atlas project, an international program for systematic exploration of the human proteome using (mono-specific) antibody-based proteomics ([43]; [www.proteinatlas.org](http://www.proteinatlas.org)). The antibody binds to an epitope located within the peptide sequence "CNEFFEGFPD" present in the C-terminal region of canine S100A4 identically encoded by all three validated transcript variants (NCBI’s accession numbers: [NM_001003161.3](https://www.ncbi.nlm.nih.gov/nuccore/NM_001003161.2), NM_001003161.3, NM_001362597.3 and [NM_001363554.1](https://www.ncbi.nlm.nih.gov/nuccore/NM_001363554.1)). Polyclonal anti-mouse IgG (H+L) antibody produced in the goat (Immunologic, Duiven, Netherlands; Poly-HRP-anti-mouse IgG (ready-to-use), catalogue number DPVM110HRP) was applied as secondary antibody for 30 min at room temperature. Staining with diaminobenzidine (DAB) produced a brownish HRP/DAB-complex. For nuclear counterstaining haematoxylin was used. In general, the complete tumour area was subjected to qIHC scoring performed as follows. The image of the stained tissue was digitised using a slide scanner (Aperio Scanscope, San Diego, California, USA) at 20-fold magnification and converted to a tagged image file format with a resolution of 1.006 µm/pixel. The percentage of tissue area positively stained with DAB was calculated in relation to the whole tissue area using a self-made script (File S2) run under the open-source platform for biological-image analysis FIJI ([44]; <https://imagej.net/Fiji)>. In brief, the tumour area was selected and its area measured. Colour deconvolution, *i.e.* unmixing using the predefined colour vectors for DAB and haematoxylin, separated the colours of the target signal and the nuclei. A bilateral filter was applied to the image showing the DAB signal for removing noise. White-intensity values of more than 200 were considered as background. Applying a particle size of at least 15 µm² reduced granular-staining artefacts.

**Statistical analysis**

Normality of transcript expression values of a stably expressed genes (SEGs) was examined by the Kolmogorov-Smirnov normality test [45]. If the null hypothesis of the test (that the distribution of data is normal) could not be rejected, outlying values were identified based on the Grubb’s test [46] run using the R package “outliers” (<https://cran.r-project.org/web/packages/outliers/index.html>). Strength and direction of the monotonic relationship between two given variables was evaluated by the Spearman's rank-order correlation coefficient *ρ* [47] that is recommended for use with data that are skewed or have outliers [48]. The strength of a linear relationship between two variables was quantified with Pearson's *r* [49]. If not otherwise indicated, statistical analysis was performed in the GraphPad Prism demo version 5 for Windows (GraphPad Software, Inc., La Jolla, CA, USA).

Logit transformation [50], one of two most common variance-stabilising transformations, was applied to the score of qIHC. Being a proportion, the score is constrained to lie between 0 and 1, and its possible variance hence depends on its mean value (more spread is possible at a mean proportion of 0.5 than at proportions of zero and one). Logit transformation calculated according to logit(*p*) = log_e_(*p*/(1-*p*)), where *p* is the proportion value of qIHC, expands the ends of the scale allowing variances at different mean values to be more naturally compared.

Observing that the normalised *S100A4* transcript levels varied almost over two orders of magnitude across the cohort of osteosarcomas, we decided to seek correlations between the transformed qIHC and the logarithm of the normalised mRNA levels. Both gene expression levels are thus subjected to a logarithmic-style transformation.

**References**

1. Thomas, R., Scott, A., Langford, C. F., Fosmire, S. P., Jubala, C. M., Lorentzen, T. D., Hitte, C., Karlsson, E. K., Kirkness, E., Ostrander, E. A., Galibert, F., Lindblad-Toh, K., Modiano, J. F. & Breen, M. (2005) Construction of a 2-Mb resolution BAC microarray for CGH analysis of canine tumors, *Genome Res.* **15**, 1831-7.

2. Thomas, R., Wang, H. J., Tsai, P. C., Langford, C. F., Fosmire, S. P., Jubala, C. M., Getzy, D. M., Cutter, G. R., Modiano, J. F. & Breen, M. (2009) Influence of genetic background on tumor karyotypes: evidence for breed-associated cytogenetic aberrations in canine appendicular osteosarcoma, *Chromosome Res.* **17**, 365-377.

3. Capes-Davis, A., Reid, Y. A., Kline, M. C., Storts, D. R., Strauss, E., Dirks, W. G., Drexler, H. G., MacLeod, R. A., Sykes, G., Kohara, A., Nakamura, Y., Elmore, E., Nims, R. W., Alston-Roberts, C., Barallon, R., Los, G. V., Nardone, R. M., Price, P. J., Steuer, A., Thomson, J., Masters, J. R. & Kerrigan, L. (2013) Match criteria for human cell line authentication: where do we draw the line?, *Int J Cancer.* **132**, 2510-9.

4. Dobin, A., Davis, C. A., Schlesinger, F., Drenkow, J., Zaleski, C., Jha, S., Batut, P., Chaisson, M. & Gingeras, T. R. (2013) STAR: ultrafast universal RNA-seq aligner, *Bioinformatics.* **29**, 15-21.

5. Trapnell, C., Pachter, L. & Salzberg, S. L. (2009) TopHat: discovering splice junctions with RNA-Seq, *Bioinformatics.* **25**, 1105-11.

6. Trapnell, C., Williams, B. A., Pertea, G., Mortazavi, A., Kwan, G., van Baren, M. J., Salzberg, S. L., Wold, B. J. & Pachter, L. (2010) Transcript assembly and quantification by RNA-Seq reveals unannotated transcripts and isoform switching during cell differentiation, *Nat Biotechnol.* **28**, 511-5.

7. Li, H., Handsaker, B., Wysoker, A., Fennell, T., Ruan, J., Homer, N., Marth, G., Abecasis, G., Durbin, R. & Proc, G. P. D. (2009) The Sequence Alignment/Map format and SAMtools, *Bioinformatics.* **25**, 2078-2079.

8. Yates, A., Akanni, W., Amode, M. R., Barrell, D., Billis, K., Carvalho-Silva, D., Cummins, C., Clapham, P., Fitzgerald, S., Gil, L., Giron, C. G., Gordon, L., Hourlier, T., Hunt, S. E., Janacek, S. H., Johnson, N., Juettemann, T., Keenan, S., Lavidas, I., Martin, F. J., Maurel, T., McLaren, W., Murphy, D. N., Nag, R., Nuhn, M., Parker, A., Patricio, M., Pignatelli, M., Rahtz, M., Riat, H. S., Sheppard, D., Taylor, K., Thormann, A., Vullo, A., Wilder, S. P., Zadissa, A., Birney, E., Harrow, J., Muffato, M., Perry, E., Ruffier, M., Spudich, G., Trevanion, S. J., Cunningham, F., Aken, B. L., Zerbino, D. R. & Flicek, P. (2016) Ensembl 2016, *Nucleic Acids Res.* **44**, D710-6.

9. Anders, S., Reyes, A. & Huber, W. (2012) Detecting differential usage of exons from RNA-seq data, *Genome Res.* **22**, 2008-2017.

10. Kolde, R. (2015) Package ‘pheatmap’ in pp. 1-8<https://cran.r-project.org/web/packages/pheatmap/pheatmap.pdf>.

11. Carmona, R., Arroyo, M., Jimenez-Quesada, M. J., Seoane, P., Zafra, A., Larrosa, R., Alche, J. D. & Claros, M. G. (2017) Automated identification of reference genes based on RNA-seq data, *Biomed Eng Online.* **16**, e65.

12. Conesa, A., Gotz, S., Garcia-Gomez, J. M., Terol, J., Talon, M. & Robles, M. (2005) Blast2GO: a universal tool for annotation, visualization and analysis in functional genomics research, *Bioinformatics.* **21**, 3674-6.

13. Fisher, R. A. (1922) On the interpretation of x(2) from contingency tables, and the calculation of P, *J R Stat Soc.* **85**, 87-94.

14. Warde-Farley, D., Donaldson, S. L., Comes, O., Zuberi, K., Badrawi, R., Chao, P., Franz, M., Grouios, C., Kazi, F., Lopes, C. T., Maitland, A., Mostafavi, S., Montojo, J., Shao, Q., Wright, G., Bader, G. D. & Morris, Q. (2010) The GeneMANIA prediction server: biological network integration for gene prioritization and predicting gene function, *Nucleic Acids Res.* **38**, W214-W220.

15. Franz, M., Rodriguez, H., Lopes, C., Zuberi, K., Montojo, J., Bader, G. D. & Morris, Q. (2018) GeneMANIA update 2018, *Nucleic Acids Res.* **46**, W60-W64.

16. Li, X., Ben-Dov, I. Z., Mauro, M. & Williams, Z. (2015) Lowering the quantification limit of the Qubit (TM) RNA HS Assay using RNA spike-in, *BMC Mol Biol.* **16**.

17. Stahlberg, A., Hakansson, J., Xian, X., Semb, H. & Kubista, M. (2004) Properties of the reverse transcription reaction in mRNA quantification, *Clin Chem.* **50**, 509-15.

18. Stahlberg, A., Kubista, M. & Pfaffl, M. (2004) Comparison of reverse transcriptases in gene expression analysis, *Clin Chem.* **50**, 1678-80.

19. Nolan, T., Hands, R. E., Ogunkolade, W. & Bustin, S. A. (2006) SPUD: a quantitative PCR assay for the detection of inhibitors in nucleic acid preparations, *Anal Biochem.* **351**, 308-10.

20. Zuker, M. (2003) Mfold web server for nucleic acid folding and hybridization prediction, *Nucleic Acids Res.* **31**, 3406-15.

21. Ye, J., Coulouris, G., Zaretskaya, I., Cutcutache, I., Rozen, S. & Madden, T. L. (2012) Primer-BLAST: a tool to design target-specific primers for polymerase chain reaction, *BMC Bioinformatics.* **13**, 134.

22. Debode, F., Marien, A., Janssen, E., Bragard, C. & Berben, G. (2017) The influence of amplicon length on real-time PCR results, *Biotechnol Agron Soc.* **21**, 3-11.

23. Brody, J. R. & Kern, S. E. (2004) Sodium boric acid: a Tris-free, cooler conductive medium for DNA electrophoresis, *Biotechniques.* **36**, 214-6.

24. Bustin, S. A., Benes, V., Garson, J. A., Hellemans, J., Huggett, J., Kubista, M., Mueller, R., Nolan, T., Pfaffl, M. W., Shipley, G. L., Vandesompele, J. & Wittwer, C. T. (2009) The MIQE guidelines: minimum information for publication of quantitative real-time PCR experiments, *Clin Chem.* **55**, 611-22.

25. Zhao, S. & Fernald, R. D. (2005) Comprehensive algorithm for quantitative real-time polymerase chain reaction, *J Comput Biol.* **12**, 1047-64.

26. Kubista, M., Sindelka, R., Tichopad, A., Bergkvist, A., Lindh, D. & Forootan, A. (2007) The Prime Technique: Real-time PCR Data Analysis, *GIT Laboratory Journal*, 33-35.

27. Vandesompele, J., De Preter, K., Pattyn, F., Poppe, B., Van Roy, N., De Paepe, A. & Speleman, F. (2002) Accurate normalization of real-time quantitative RT-PCR data by geometric averaging of multiple internal control genes, *Genome Biol.* **3**, RESEARCH0034.

28. Hellemans, J., Mortier, G., De Paepe, A., Speleman, F. & Vandesompele, J. (2007) qBase relative quantification framework and software for management and automated analysis of real-time quantitative PCR data, *Genome Biol.* **8**, R19.

29. Andersen, C. L., Jensen, J. L. & Orntoft, T. F. (2004) Normalization of real-time quantitative reverse transcription-PCR data: A model-based variance estimation approach to identify genes suited for normalization, applied to bladder and colon cancer data sets, *Cancer Res.* **64**, 5245-5250.

30. Pfaffl, M. W., Tichopad, A., Prgomet, C. & Neuvians, T. P. (2004) Determination of stable housekeeping genes, differentially regulated target genes and sample integrity: BestKeeper - Excel-based tool using pair-wise correlations, *Biotechnol Lett.* **26**, 509-515.

31. Silver, N., Best, S., Jiang, J. & Thein, S. L. (2006) Selection of housekeeping genes for gene expression studies in human reticulocytes using real-time PCR, *BMC Mol Biol.* **7**, e33.

32. Xie, F., Xiao, P., Chen, D., Xu, L. & Zhang, B. (2012) miRDeepFinder: a miRNA analysis tool for deep sequencing of plant small RNAs, *Plant Mol Biol*.

33. Pihur, V., Datta, S. & Datta, S. (2009) RankAggreg, an R package for weighted rank aggregation, *BMC Bioinformatics.* **10**, 62.

34. Obayashi, T., Kagaya, Y., Aoki, Y., Tadaka, S. & Kinoshita, K. (2019) COXPRESdb v7: a gene coexpression database for 11 animal species supported by 23 coexpression platforms for technical evaluation and evolutionary inference, *Nucleic Acids Res.* **47**, D55-D62.

35. The Gene Ontology, C. (2019) The Gene Ontology Resource: 20 years and still GOing strong, *Nucleic Acids Res.* **47**, D330-D338.

36. Yeh, H. S. & Yong, J. (2016) Alternative Polyadenylation of mRNAs: 3'-Untranslated Region Matters in Gene Expression, *Mol Cells.* **39**, 281-5.

37. Liu, H., Han, H., Li, J. & Wong, L. (2005) DNAFSMiner: a web-based software toolbox to recognize two types of functional sites in DNA sequences, *Bioinformatics.* **21**, 671-3.

38. Green, M. R. & Sambrook, J. (2019) Rapid Amplification of Sequences from the 3' Ends of mRNAs: 3'-RACE, *Cold Spring Harb Protoc.* **2019**, pdb prot095216.

39. Sanders, R., Mason, D. J., Foy, C. A. & Huggett, J. F. (2013) Evaluation of digital PCR for absolute RNA quantification, *PLoS One.* **8**, e75296.

40. Majumdar, N., Banerjee, S., Pallas, M., Wessel, T. & Hegerich, P. (2017) Poisson Plus Quantification for Digital PCR Systems, *Sci Rep.* **7**, 9617.

41. Seyed Jafari, S. M. & Hunger, R. E. (2017) IHC Optical Density Score: A New Practical Method for Quantitative Immunohistochemistry Image Analysis, *Appl Immunohistochem Mol Morphol.* **25**, e12-e13.

42. Jensen, K., Krusenstjerna-Hafstrom, R., Lohse, J., Petersen, K. H. & Derand, H. (2017) A novel quantitative immunohistochemistry method for precise protein measurements directly in formalin-fixed, paraffin-embedded specimens: analytical performance measuring HER2, *Modern Pathol.* **30**, 180-193.

43. Thul, P. J. & Lindskog, C. (2018) The human protein atlas: A spatial map of the human proteome, *Protein Sci.* **27**, 233-244.

44. Schindelin, J., Arganda-Carreras, I., Frise, E., Kaynig, V., Longair, M., Pietzsch, T., Preibisch, S., Rueden, C., Saalfeld, S., Schmid, B., Tinevez, J. Y., White, D. J., Hartenstein, V., Eliceiri, K., Tomancak, P. & Cardona, A. (2012) Fiji: an open-source platform for biological-image analysis, *Nat Methods.* **9**, 676-82.

45. Massey, F. J. (1951) The Kolmogorov-Smirnov Test for Goodness of Fit, *J Am Stat Assoc.* **46**, 68-78.

46. Grubbs, F. E. (1969) Procedures for Detecting Outlying Observations in Samples, *Technometrics.* **11**, 1-&.

47. Spearman, C. (1904) The proof and measurement of association between two things, *Am J Psychol* **15**, 72–101.

48. Zou, K. H., Tuncali, K. & Silverman, S. G. (2003) Correlation and simple linear regression, *Radiology.* **227**, 617-22.

49. Pearson, K. (1896) III. Regression, heredity and panmixia in *Mathematical contributions to the theory of evolution*  pp. 253–318.

50. Berkson, J. (1944) Application of the Logistic Function to Bio-Assay, *J Am Stat Assoc.* **39**, 357-365.
